# The Role of Nerve Growth Factor in Maintaining Proliferative Capacity, Colony‐Forming Efficiency, and the Limbal Stem Cell Phenotype

**DOI:** 10.1002/stem.2921

**Published:** 2018-12-31

**Authors:** Sai Kolli, Sanja Bojic, Ali E. Ghareeb, Marzena Kurzawa‐Akanbi, Francisco C. Figueiredo, Majlinda Lako

**Affiliations:** ^1^ Newcastle University, Institute of Genetic Medicine Newcastle upon Tyne United Kingdom; ^2^ University Hospital Birmingham NHS Foundation Trust Birmingham United Kingdom; ^3^ Department of Ophthalmology Royal Victoria Infirmary Newcastle upon Tyne United Kingdom

**Keywords:** Limbal stem cells, Nerve growth factor, Proliferation, Colony‐forming efficiency, Limbal stem cell markers, Stem cell niche, Corneal epithelium, Limbal stem cell deficiency

## Abstract

Nerve growth factor (NGF) has demonstrated great benefit in the treatment of neurotrophic corneal ulcers. There is evidence for multiple modes of action in promoting corneal healing, but only indirect evidence exists for NGF's effects on limbal stem cells (LSCs). Understanding the role of NGF in LSC biology will improve our understanding of paracrine regulation of the limbal niche and the design of stem cell‐based therapies for conditions such as LSC deficiency. In this article, we studied the regulation of NGF signaling components during LSC differentiation and the role of NGF in LSC proliferation and maintenance of the stem cell phenotype. LSC differentiation was induced by prolonged (40 day) culture which resulted in a significant increase in cell size, decrease in colony‐forming efficiency and expression of putative LSC markers. A protein microarray measuring expression of 248 signaling proteins indicated the low affinity NGF receptor p75^NTR^ to be the most downregulated protein upon differentiation. Further confirmation by Western blotting and real‐time quantitative polymerase chain reaction indicated that NGF and p75^NTR^ are expressed in early LSC cultures and downregulated upon differentiation. LSC cultures grown in the presence of anti‐NGF antibody showed decreased colony‐forming efficiency, DNA replication and expression of putative LSC markers *ABCG2* and *C/EBPδ*. Supplementation of LSC culture medium with NGF extended the life span of LSC cultures in vitro and increased the expression of putative LSC markers Δ*Np63α* and *ABCG2*. Taken together, our data indicate that NGF signaling is a key promoter of LSC proliferation, colony‐forming efficiency, and a maintainer of the LSC phenotype. stem cells *2019;37:139–149*

**Significance Statement**

Nerve growth factor (NGF) has demonstrated healing properties in numerous ocular surface diseases, in particular neurotrophic corneal ulcers. Understanding the effects of NGF on the stem cells of the cornea (limbal stem cells [LSCs]) will lead to better treatments for debilitating corneal diseases. Using NGF‐blocking antibodies and recombinant human NGF, results of this study show that NGF promotes the LSC characteristics of colony formation, proliferation and expression of stem cell markers. Therefore, NGF promotes “stemness” in corneal epithelium and these properties may be utilized in improving LSC transplant and developing topical therapeutic agents.

## Introduction

The cornea is the transparent structure and main refractive surface of the eye 1. It is composed of three main layers: an outer stratified epithelium, stroma, and an inner single‐cell layered endothelium. The terminally differentiated epithelial cells of the cornea are derived from limbal stem cells (LSCs), which reside in the basal epithelium of the Palisades of Vogt found at the corneoscleral limbus 2, 3, 4, 5, 6, 7, 8.

Corneal transparency is essential for vision. However, it can be degraded by direct insults such as infection, trauma, and chemical burns. Numerous cytokines including vascular endothelial growth factor, epidermal growth factor, and keratinocyte growth factor have been implicated in regulation of the corneal healing process 9, 10, 11, 12, 13, 14, 15, 16. Nerve growth factor (NGF) is a member of the neurotrophin family, and is constitutively expressed in the basal limbus with lower expression in the basal corneal epithelium 17, 18, 19, 20, 21. Its high‐affinity receptor, TrkA, has the same pattern of expression 18, 19, 21, 22. The nonspecific neurotrophin receptor p75^NTR^ is expressed in the basal epithelium of the limbus, peripheral, and central cornea 18. The NGF precursor, pro‐NGF, has high affinity for p75^NTR^ but has minimal activity with TrkA receptors 23, 24.

In vitro and in vivo studies demonstrated that NGF inhibits apoptosis and inflammation in a model of the diabetic cornea 25, promotes corneal epithelial migration 26, and that blocking of NGF activity inhibits expansion of limbal epithelium on amniotic membrane 21. Furthermore, clinical trials have shown that topical application of NGF improves corneal healing in neurotrophic ulcers 27, inflammatory conditions 28, and following cataract surgery 29. Together, these studies show that NGF is a pleiotropic factor, stimulating corneal healing through several putative mechanisms.

In limbal stem cell deficiency (LSCD), the corneal epithelium is not replenished sufficiently, inhibiting corneal healing. Furthermore, the protective barrier function of the limbus is lost, and conjunctivalization, vascularization, and inflammation of the cornea occur. Ultimately, the transparency of the cornea is degraded and vision impaired 1. Improved understanding of the local factors which regulate the LSC niche will aid in the development of new treatments for this debilitating disease.

Despite its efficacy in the treatment of neurotrophic ulcers and its potential in the treatment of several other corneal diseases including LSCD, the role of NGF in the maintenance of LSCs remains poorly understood. In this study, we investigated the expression of NGF and its receptors in ex vivo expansion and differentiation of LSCs and assessed its role in LSC proliferation and potency.

## Materials and Methods

### Human Donor Tissue

The human limbal tissue of nine donors was provided by the NHS Blood and Transplant Eye Banks and had been specifically donated for use in research. Ethical approval for using human corneal tissue was sought and granted prior to this study.

### Culture of Human Limbal Epithelium

In brief, LSCs harvested from cadaveric corneoscleral rims were cocultured on mitotically inactivated, murine 3T3‐fibroblast feeder layers as described in Ahmad et al. 30. The epithelial medium was renewed on the third day to allow limbal cell adherence to the culture flask, and then every other day thereafter. For subculture, 3T3‐fibroblasts were removed by incubating the culture with 0.02% EDTA solution (Sigma‐Aldrich, St.Louis, MO, US, https://www.sigmaaldrich.com). The EDTA was removed and the culture incubated with 0.05% trypsin for 5 minutes to release the limbal epithelial cells. The cell suspension was diluted with epithelial medium and centrifuged as above. The resulting cell pellet was resuspended in epithelial medium and 10,000–12,000 viable cells from this suspension were then placed in a 9.6 cm^2^ culture well previously plated with mitotically inactivated 3T3 cells.

### Differentiation of Human Limbal Epithelium

**Figure 1:**
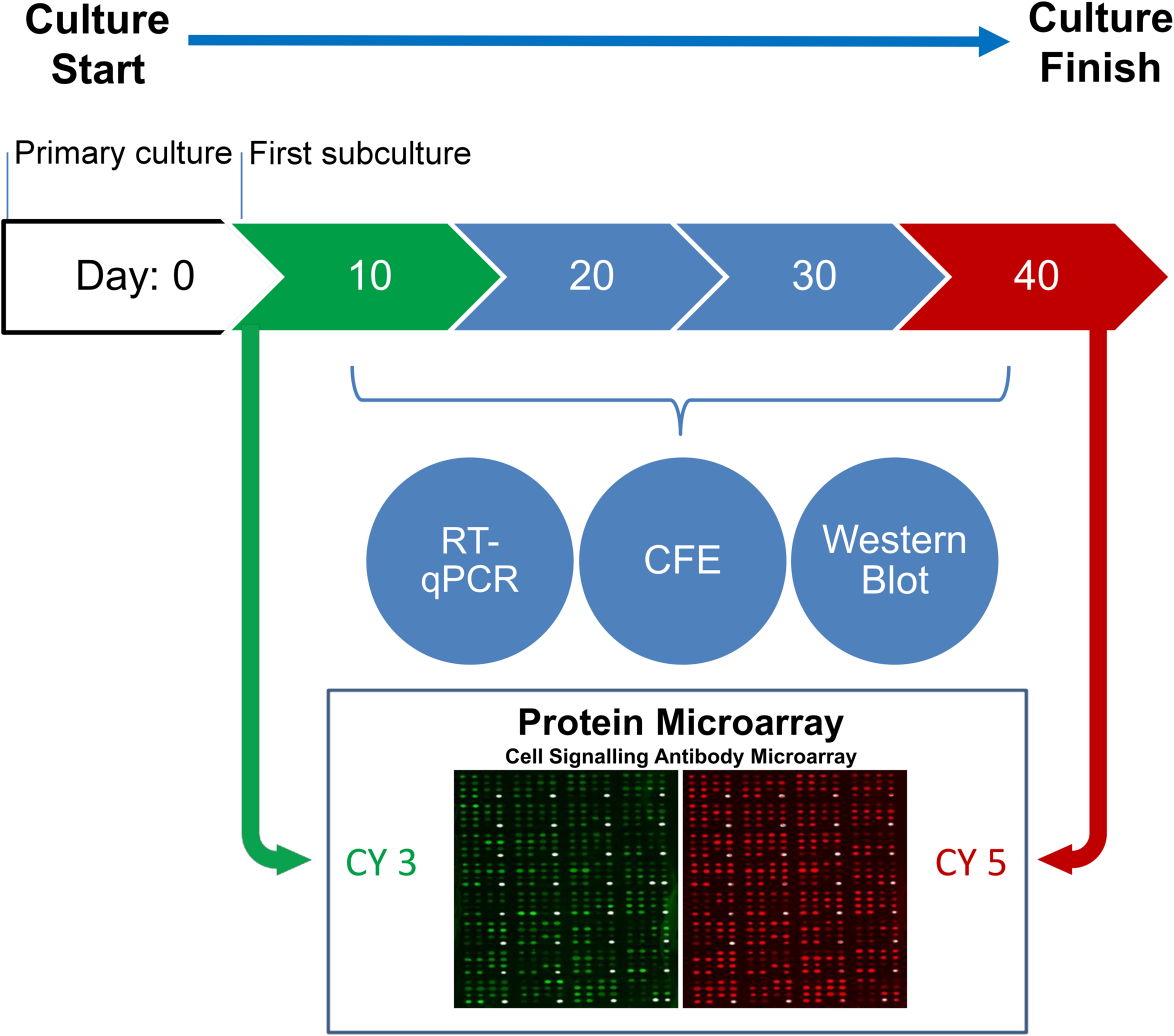
Schematic representation of corneal epithelial stem cell differentiation protocol and sample collection time points. Abbreviations: RT‐qPCR, real‐time quantitative polymerase chain reaction; CFE, colony‐forming efficiency.

The study protocol is summarized in Figure 1. At day 10 after initiation of the culture colony‐forming efficiency (CFE), real‐time quantitative polymerase chain reaction (RT‐qPCR) and protein array‐based analysis was carried out. Some of the wells were used to subculture the cells which were allowed to reach confluence and differentiate for a further 30 days. Samples were collected at days 20, 30, and 40 for CFE, RT‐qPCR, and protein assays. Cell size was measured at each time point using Axiovert software (Zeiss Axiovision, Oberkochen, Germany, https://www.zeiss.com). Color photographs were taken every 10‐day interval using a Zeiss microscope at ×10 magnification.

### Protein Microarray of Undifferentiated and Differentiated Limbal Cultures

Panorama Antibody Microarray for Cell signaling kits containing 248 different antibodies spotted in duplicate on nitrocellulose coated glass (Sigma‐Aldrich, St.Louis, MO, US, https://www.sigmaaldrich.com) were used for this analysis. One milligram of day 10 and day 40 cell extracts was collected, labeled with Cy3 and Cy5 respectively, and hybridized to the slides according to manufacturer's instructions. The Cy3 and Cy5 signals were read on Gene Pix Pro 4.0 (Molecular Devices, Union City, CA, US, http://www.moleculardevices.com). Sample processing was carried out using three normalization steps which involved dye swap where necessary, dividing each spot by the control channel, and normalizing each spot to the 50th centile of the entire chip. Enriched proteins were those with a fold‐change of ≥1.2. The enriched proteins were mapped to corresponding coding genes and analysis of gene ontology by biological process was performed using Enrichr with terms ranked by combined *p* value and *Z* score 31, 32. Enriched pathways were identified from the Reactome pathway database 33, 34 and visualizations produced using ReactomePA 35, a Bioconductor package for R, the statistical programming language 36.

### NGF Addition to Limbal Epithelial Cell Culture

LSC cultures at 50%–60% confluence were exposed to recombinant human β‐NGF (rhNGF) (PeproTech, Rocky Hill, NJ, http://www.peprotech.com) at a final concentration of 100 ng/ml. Cells were fed every other day for 40 days. After harvesting, the cells were used for RT‐qPCR analysis. Additionally, LSC were passaged until senescence in control and NGF supplemented conditions to further examine the role of NGF in proliferation and colony forming ability of LSCs.

### NGF Blocking in Limbal Epithelial Cell Culture

Anti‐human NGF antibody (R&D systems, MAB256, Minneapolis, MN, US, http://www.rndsystems.com) was used at a concentration of 5 μg/ml in the culture wells with 50%–60% confluent limbal epithelial cultures. Measured by its ability to neutralize β‐NGF‐induced proliferation in the TF‐1 human cell line, the Neutralization Dose (ND_50_) is typically 0.1–0.4 μg/ml in the presence of 5 ng/ml recombinant human β‐NGF 37. We used a dose (5 μg/ml) which resulted in complete blocking of NGF‐induced proliferation in the TF‐1 model. In order to examine its effect, cells were exposed to anti‐NGF for the period of 14 days and prolonged period of 40 days.

### CFE Assays

Mitotically inactivated 3T3 fibroblasts in 3T3 medium were plated in a 9.6 cm^2^ tissue culture well (Scientific Laboratory Supplies, Nottingham, UK, https://www.scientificlabs.co) at a density of 2.4 × 10^4^/cm^2^ and placed in a tissue culture incubator overnight to allow the establishment of a 3T3 feeder layer. The following day, up to 1,000 viable cells from LSC cultures at day 14 of in vitro culture were plated onto the prepared 3T3 cells together with 2 ml of epithelial medium. The CFE culture was then placed in the tissue culture incubator and the epithelial medium was changed on the third day and then every second day thereafter. The CFE was measured on the 12th day of the culture. This was performed by removal of the epithelial medium followed by two brief irrigations with phosphate buffered solution (PBS). The culture was then fixed with 3.7% formaldehyde (VWR, Brooklyn, NY, US, https://www.vwr.com) in PBS for 10 minutes at room temperature. The formaldehyde solution was removed, and the culture was irrigated with PBS. The colonies were stained by incubation with 1% Rhodamine B (Sigma‐Aldrich, St.Louis, MO, US, https://www.sigmaaldrich.com) in methanol (VWR) for 10 minutes at RT. Following staining, the colonies were counted. The CFE was then calculated as:

number of colonies formed/number of cells plated×100.

### Real‐Time Quantitative Polymerase Chain Reaction

RNA was extracted using the Promega tissue extraction kit [Promega, Madison, WI, US, http://www.promega.com) as per the manufactures instructions. One microgram of RNA was reverse transcribed using random primers (Promega, Madison, WI, US, http://www.promega.com). RT‐qPCR was performed using a Quant Studio 7 Flex system (Applied Biosystems, Foster City, CA, http://www.appliedbiosystems.com) with SYBR Green (Promega, Madison, WI, US, http://www.promega.com). The expression of the putative LSCs markers, Δ*Np63α*, *ABCG2*, and *C/EBPδ* and the corneal differentiation marker *CK3* was measured as well as the expression of NGF and its receptors, TrkA and p75^NTR^. Each primer (listed in Supporting Information Table S1) was used at a concentration of 1 μM, and at a ratio of 50:50 for forward and reverse. The reaction parameters were as follows: 95°C for 15 minutes to denature the cDNA and primers, 40 cycles of 94°C for 15 seconds followed by primer specific annealing temperature for 30 seconds, succeeded by a melt curve. A comparative Ct method was used to calculate the levels of relative expression, whereby the Ct was normalized to the endogenous control (glyceral‐dehyde‐3‐phosphate dehydrogenase). This calculation gives the ΔCt value, which was then normalized to a reference sample (i.e., a positive control), giving the ΔΔCt. The fold change was calculated using the following formula: 2^‐ΔΔCt^. Statistical significance analyses were evaluated by using Prism (GraphPad, La Jolla, CA, US, https://www.graphpad.com).

### Western Blotting

Confluent cell cultures grown on six well plates were placed on ice. The cells were scraped and washed three times with ice‐cold PBS. After removing the supernatant at the last wash, one hundred microliters of RIPA buffer (Merck, Kenilworth, NJ, US, https://www.merckgroup.com) was added to each well's pellet and was left in situ for 40 minutes. Following the incubation cells were sonicated briefly two times for 2–3 seconds and finally centrifuged at 1000*g* for 10 minutes at 4°C. The supernatant was used in subsequent assays. Protein concentration was determined using the bicinchoninic assay (Pierce BCA Protein Assay Kit, ThermoFisher, Voltam, MA, US, https://www.thermofisher.com). A total to 37 μg of protein was prepared for Western blotting by mixing with the loading dye (NuPAGE LDS Sample Buffer 4×, ThermoFisher Voltam, MA, US, https://www.thermofisher.com) and NuPAGE Sample Reducing Agent 10× (ThermoFisher Voltam, MA, US, https://www.thermofisher.com), and heating at 70°C for 10 minutes. Protein samples were then resolved in NuPAGE 4%–12% Bis‐Tris protein gels by electrophoresis in MES running buffer (ThermoFisher Voltam, MA, US, https://www.thermofisher.com) alongside the SeeBlue Plus2 Pre‐stained protein standard (ThermoFisher Voltam, MA, US, https://www.thermofisher.com). Gels were transferred to polyvinylidene fluoride (PVDF) membranes using the iBlot2 gel transfer device and compatible iBlot 2 PVDF gel transfer stacks (ThermoFisher Voltam, MA, US, https://www.thermofisher.com). Protein loading was visualized using Ponceau S staining (Sigma, St.Louis, MO, US, https://www.sigmaaldrich.com) and subsequently de‐stained in distilled water and Tris Buffered Saline‐0.1% Tween 20 (TBST). Membranes were blocked in 5% nonfat milk in TBST for 1 hour at room temperature. Antibodies used in the Western blot are detailed in Supporting Information Table S2. Primary antibodies were diluted in 5% nonfat milk—TBST or 5% Bovine Serum Albumin (BSA)—TBST and incubated with the membranes over night at 4°C with agitation. Membranes were washed in TBST three times for 10 minutes and incubated with appropriate secondary antibodies at 1:2,000 dilution in 5% nonfat milk—TBST for 1 hour at room temperature (Dako, Agilent Pathology Solutions, Santa Clara, CA, US, https://www.agilent.com, UK). Membranes were washed in TBST at least three times for 10 minutes and developed using Pierce ECL Plus Western Blotting Substrate (ThermoFisher, Voltam, MA, US, https://www.thermofisher.com). Chemiluminescent signal was detected using the Amersham Imager 600 (GE Healthcare, Chicago, IL, US, https://www.gehealthcare.com, US).

### Assays of Proliferation and Apoptosis

Proliferation in the standard culture system was compared to standard culture with anti‐NGF antibody using the Click‐IT EdU flow cytometry proliferation assay (ThermoFisher, Voltam, MA, US, https://www.thermofisher.com) according to the manufacturer's guidelines. In brief, the cells of interest were incubated in tissue culture medium at optimal conditions. EdU (5‐ethynyl‐2′‐deoxyuridine) was added at a concentration of 10 μM and incubated for various periods under the standard incubation parameters. The cells were then harvested and washed with 1% BSA in PBS and centrifuged at 500*g* for 5 minutes after which the supernatant was removed and the procedure repeated. A 100 μl of Click‐iT fixative was added to the pellet in the flow tube and mixed and kept in the dark for 15 minutes at RT. After one further wash, the cells were permeabilized with the Click‐iT permeabilization reagent. Then, 0.5 ml of Click‐iT reaction cocktail was added and the tube incubated at RT in the dark for 30 minutes. The cells were washed, pelleted and resuspended in permeabilization and wash reagent ready for EdU detection on the flow cytometer at 404 nm excitation and 450 nm emission.

Apoptosis was measured in standard culture and NGF blocked cultures by APO‐DIRECT assay (BD biosciences, San Diego, CA, US, http://www.bdbiosciences.com) according to the manufacturer's guidelines. In brief, the cells were first fixed by suspension in 1% paraformaldehyde in PBS (pH 7.4) at a concentration of 1–2 × 10^6^ cells per milliliter and placed on ice for 30–60 minutes. The cells were then centrifuged after which the supernatant was removed and the cells were repeatedly washed in PBS. The cells were then adjusted to a concentration of 1–2 × 10^6^ cells per milliliter in 70% ice‐cold ethanol and allowed to stand for a minimum of 30 minutes on ice. Following fixation, the cells were then stained according to the BD protocol. The staining procedure was performed for negative and positive controls also. Analysis was performed on a flow cytometer equipped with a 488 nm argon laser light source with two dyes used: propidium iodide to stain total DNA and Fluorescein‐Deoxyuridine Triphosphate (FITC‐dUTP) to stain apoptotic cells.

### Statistical Analysis

The statistical package SPSS (Version 24.0. IBM Corp., Armonk, NY, US, https://www.ibm.com) was used to compute statistical tests. Pairwise Student's *t* test was used for anti‐NGF and NGF addition experiments. One‐way analysis of variance was used for all other experiments. Tukey's honest significant difference was used for post hoc, between group analyses. A *p* value of <.05 was considered significant.

## Results

### Corneal Epithelial Stem Cell Differentiation

LSCs formed tight compact epithelial‐like colonies when cultured with mitotically inactivated mouse fibroblast 3T3 cells (Supporting Information Fig. S1A, S1B, and S1C). Upon reaching confluence and continuous culture, cells displaying the morphological features of differentiated corneal epithelial cells were observed with low nucleus:cytoplasm ratio, morphological heterogeneity, and large cell surface area (Supporting Information Fig. S1C–S1G). Cell surface area increased significantly with time from initiation of the culture (*p* < 1 × 10^−16^). From day 0 to day 40 after initiation of the culture, there was an eightfold increase in cell surface area (Supporting Information Fig. S1D–S1H) that suggests LSC differentiation.

**Figure 2:**
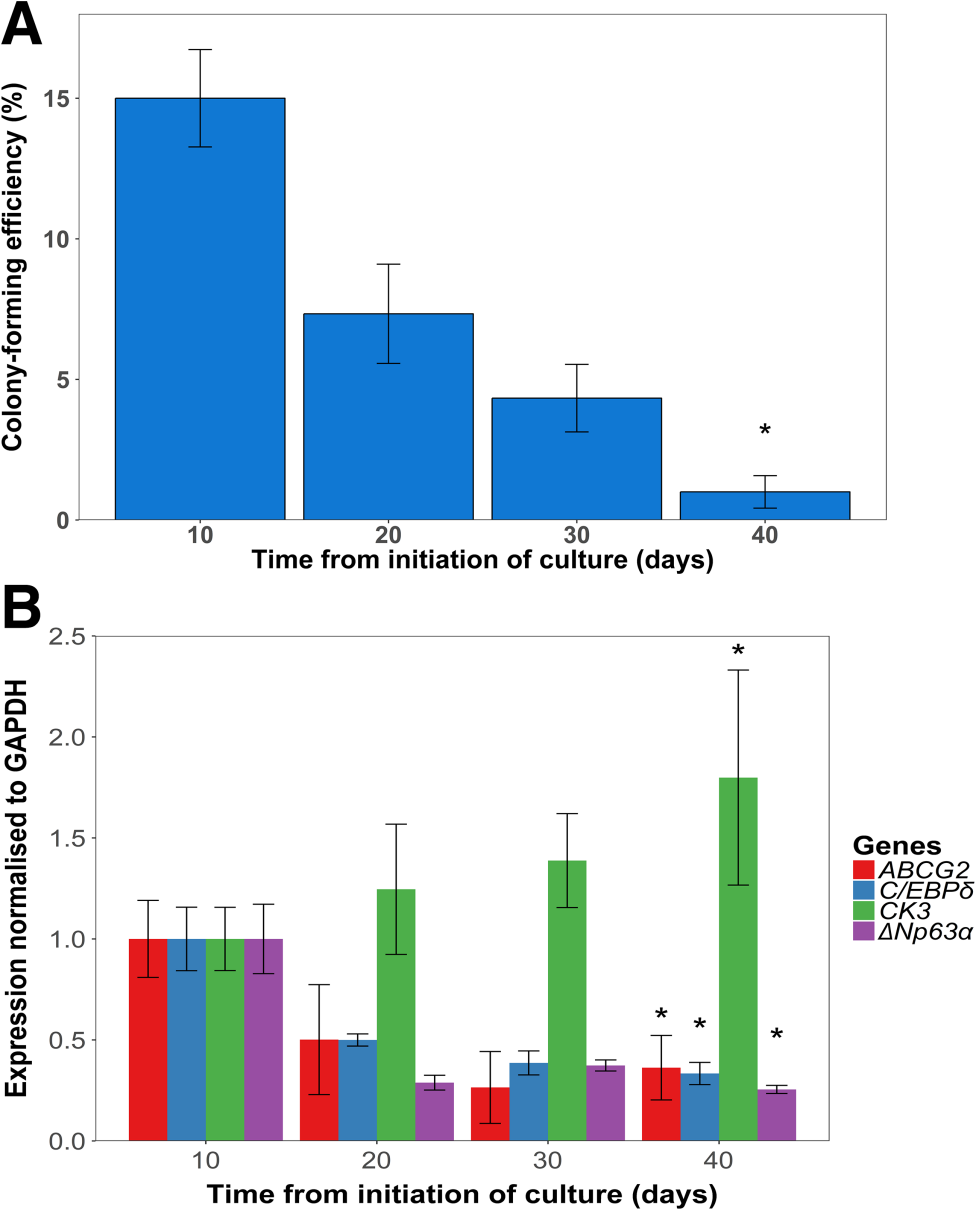
Limbal stem cells (LSC) differentiation results in reduced colony‐forming efficiency (CFE), reduced expression of putative LSC markers, and increased expression of putative markers of differentiated corneal epithelium. **(A):** Bar chart showing the results of the CFE assay during differentiation protocol. There is a significant reduction in CFE during the differentiation protocol (*p* < .001; *n* = 3; one‐way analysis of variance (ANOVA); SEM shown by error bars). **(B):** Real‐time quantitative polymerase chain reaction (RT‐qPCR) of putative LSC markers and a corneal differentiation marker at different stages after primary culture. There is a statistically significant decrease in putative LSC markers *ABCG2*, *C/EBPδ*, Δ*Np63α* (*p* < .000001, <.001, and < .000001, respectively; *n* = 3; one‐way ANOVA; SEM) and an increase in differentiation marker *CK3* throughout the culture process (*p* < .001; *n* = 3; one‐way ANOVA; SEM). Between‐group analyses are shown in Supporting Information Table S4. Abbreviation: GAPDH, glyceral‐dehyde‐3‐phosphate dehydrogenase.

CFE decreased significantly with time from culture initiation (*p* < .001) with a CFE of 1% at the end of the differentiation protocol (Fig. 2A).

Gene expression analysis showed an increase in the expression of corneal differentiation markers and a decrease in putative LSC markers phenotype with time from culture initiation (Fig. 2B). *ABCG2*, *C/EBPδ*, and Δ*Np63α* mRNA levels decreased significantly with time after initiation of culture (*p* < .000001, <.001, <.000001, respectively), whereas *CK3* expression increased significantly (*p* < .001).

### Protein Microarray‐Based Expression Study

**Figure 3:**
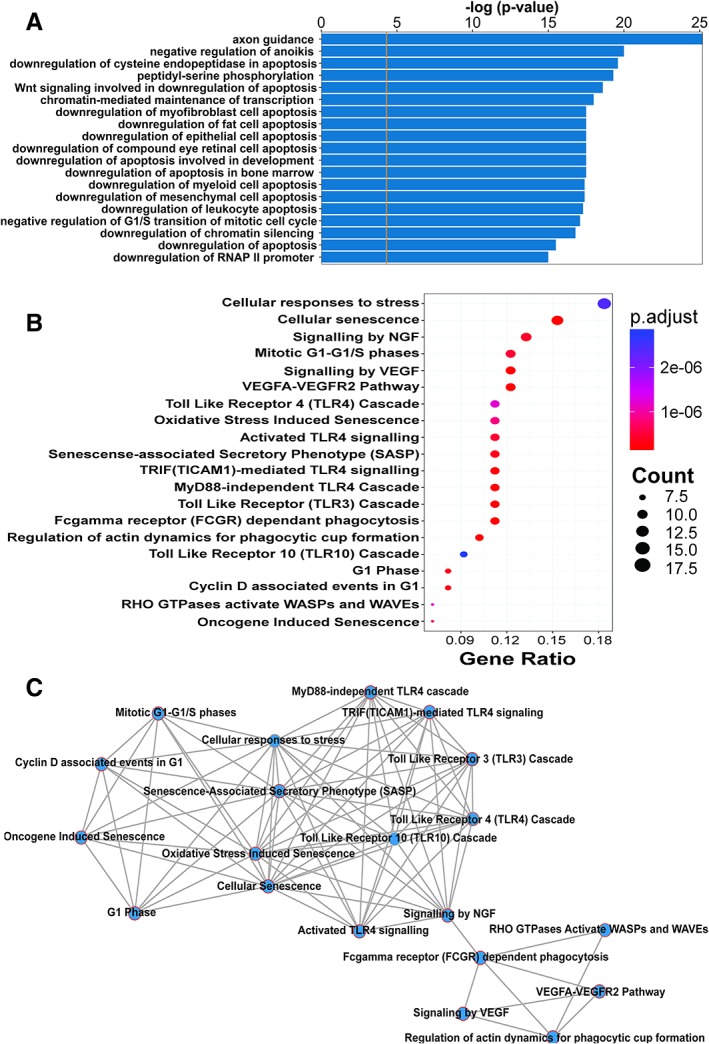
Bioinformatics analysis of differentially expressed proteins between the stem cell and differentiated states shows that cell cycle regulation and apoptosis are enriched biological process terms. NGF signalling is a highly enriched pathway term and potentially controls several other pathways known to be involved in corneal healing. **(A)** Top 20 ranked biological functions by combined *p*‐value and *Z*‐score (Enrichr) against corresponding negative log *p*‐values. The orange line represents a *p*‐value = 0.05. A list of genes coding for the proteins analysed in the Panorama Cell Signalling Protein microarray was drawn up. Genes which met the 1.2‐fold change cut‐off were used in the analysis of gene ontology. **(B)** Dot‐plot representing the significance of the top 20 enriched pathways ranked by number of genes involved (ReactomePA). Genes are clustered into pathway terms based on membership of Reactome pathways. Count is the number of genes involved in the pathway. Gene Ratio is the ratio of the genes involved in the pathway to the total number of genes analysed. P adjust is the adjusted *p*‐value. (C) Functional enrichment map of the top 20 enriched pathways by number of genes involved (ReactomePA). Nodes represent Reactome pathway terms. Edges between pathway terms represent overlap of pathway members.

Microarray analysis of the expression levels of 248 signaling protein on day 10 and day 40 of the differentiation protocol demonstrated differential expression of 109 signaling proteins with a fold‐change ≥1.2, of which 65 were upregulated and 44 downregulated upon differentiation of our LSC cultures. NGF receptor p75^NTR^ was the protein with the greatest fold‐change in expression between the LSCs and differentiated phenotypes, with expression decreasing 2.77‐fold upon differentiation (Supporting Information Table S3). Gene ontology analysis of biological processes revealed that these proteins were involved in processes such as inhibition of apoptosis, cell cycle regulation, and regulation of transcription. Axon guidance was the most statistically significant term. Regulators of programmed cell death were particularly prevalent, forming 13 of the top 20 hits in the gene ontological analysis. Interestingly, hits 14 and 16 were downregulation of retinal cell apoptosis and epithelial cell apoptosis, respectively (*p* < .00001 and <.00001). The top 20 biological processes identified by Enrichr are shown in Figure 3A, ranked by *p* value.

Analysis of differentially expressed signaling pathways shows that pathways involved in NGF and VEGF signaling, Toll‐like receptor cascades, and the cell cycle are very significantly enriched. The top 20 most significantly enriched pathways are shown in Figure 3B, ranked by the number of proteins involved. Known important downstream effectors of corneal NGF signaling are represented here (see the Discussion section). NGF signaling is the third most prominently enriched pathways in terms of the number of genes involved, and its enrichment is highly significant, as shown in Figure 3B. A network representation of putative interactions between these pathways is shown in Figure 3C. NGF signaling is alone atop the hierarchy of interactions and is notably the only pathway term which connects the small module containing VEGF signaling to the larger module containing Toll‐like receptor, cellular senescence, and the cell cycle pathways.

### Western Blot of NGF and Its Receptors

**Figure 4:**
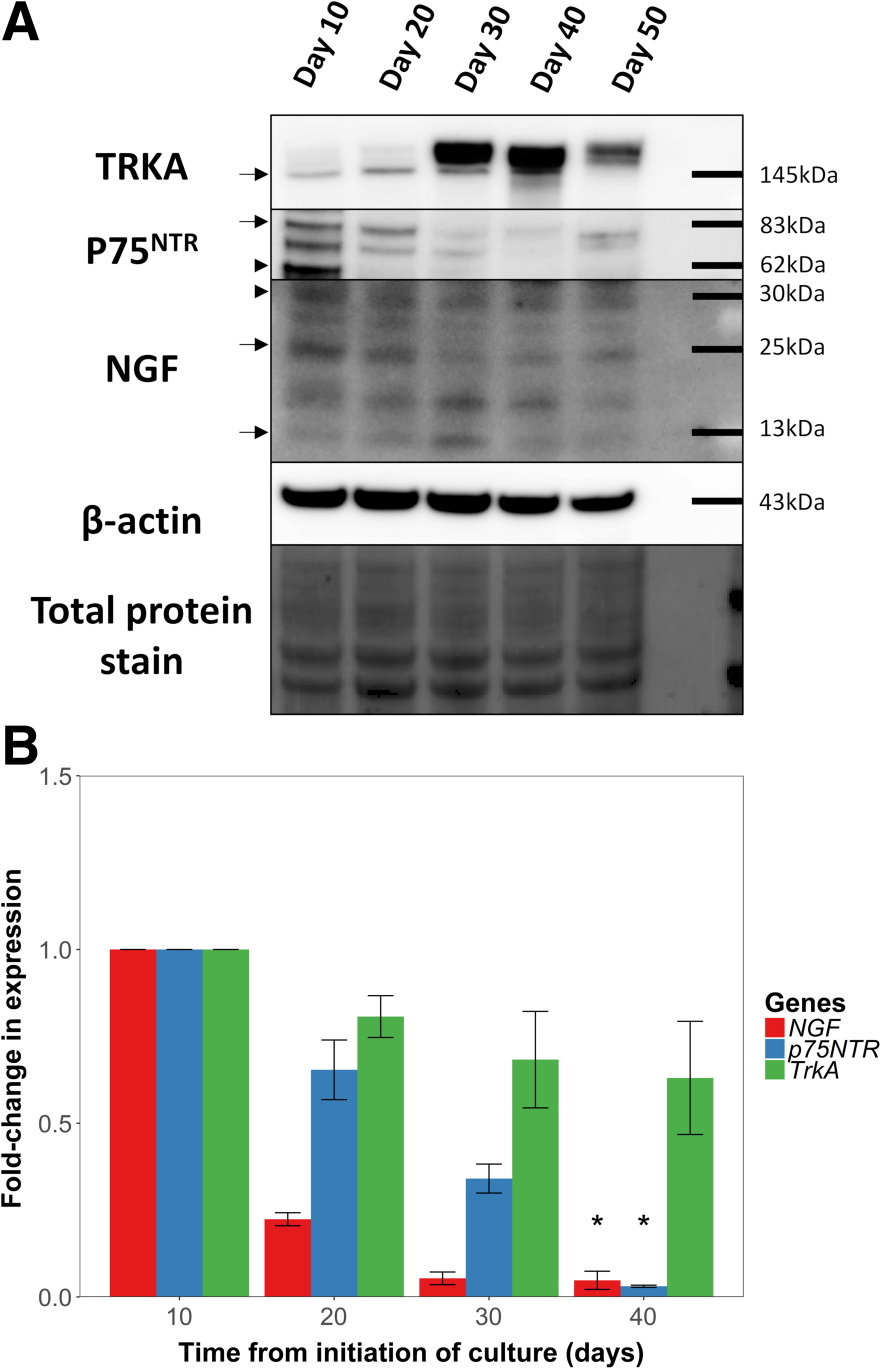
Expression of nerve growth factor (NGF) and its receptors during the differentiation of limbal stem cells. **(A):** Western blot analysis of TrkA, p75^NTR^, NGF, with β‐actin (43 kDa) as a housekeeping control at days 10, 20, 30, 40, and 50 of culture. Mature NGF was detected as two bands at 27 and 13 kDa. Two bands likely representing two glycosylation forms of p75^NTR^ at 62 and 83 kDa were also found as well as a band at 145 kDa representing TrkA. p75^NTR^ and NGF decreased with differentiation while TrkA expression was variable with no clear trend. Full blots are shown in Supporting Information Figure S2A. **(B):** RT‐qPCR of NGF, p75^NTR^, and TrkA at days 10, 20, 30, and 40 of culture showing a significant decrease of NGF and p75^NTR^ during differentiation (*p* < 1 × 10^−9^ and *p* < 1 × 10^−5^, respectively).

To further investigate the expression of NGF pathway components that were not included in the protein microarray and confirm the array‐based results, we used Western immunoblotting (Fig. 4A and Supporting Information Fig. S2Ai–S2Av). The presence of NGF and its receptors TrkA and p75^NTR^ was confirmed by Western blot analysis using β‐actin (43 kDa) as a housekeeping control. Qi et al. reported pro‐NGF at 30 kDa and two bands of weaker intensity at 25 and 14 kDa (believed to be mature forms of NGF) to be present in limbal epithelium 38. Similarly, we found one band around 30 kDa likely representing pro‐NGF (Fig. 4A, arrowhead) and two additional bands at 25 and 13 kDa likely representing mature NGF (Fig. 4A, arrows). Two bands likely representing the short variant (62 kDa, Fig. 4A, arrowhead) and the full‐length variant (83 kDa, Fig. 4A, arrow) of p75^NTR^ were also found, which is corroborated by data published by von Schack et al. 39. Moreover, the band representing TrkA was detected at 145 kDa (Fig. 4A, arrow**)**, corroborating previously published data by Ríos et al. in conjunctival cells 40.

NGF and its receptors were all expressed in LSC cultures early in the differentiation protocol (day 10). With each successive 10‐day interval, the expression of all forms of NGF and its low‐affinity nonspecific receptor p75^NTR^ (both its short and full length form) decreased. On the other hand, expression of NGF's high affinity receptor, TrkA, was variable throughout the culture period. In summary, these data suggest that NGF and p75^NTR^ are expressed in LSCs and downregulated during the differentiation process.

### RT‐qPCR of NGF and Its Receptors

We validated our findings from the Western blot experiments with a gene expression study. Using RT‐qPCR, we measured the expression of *NGF*, *TrkA*, and *p75* ^*NTR*^ at 10‐day intervals through a culture period of 40 days in standard culture conditions (Fig. 4B; for between group analysis, see Supporting Information Table S4).

The expression levels of *NGF* and *p75* ^*NTR*^ decrease markedly with time in standard culture conditions (*p* < 1 × 10^−9^ and *p* < 1 × 10^−5^, respectively). The expression of *TrkA* decreases slightly but this decrease is not significant ( = .168). These results are concordant with the results of the Western Blot of NGF, TrkA, and p75^NTR^.

### Blocking of NGF Action through a Neutralizing Antibody Results in LSC Differentiation

**Figure 5:**
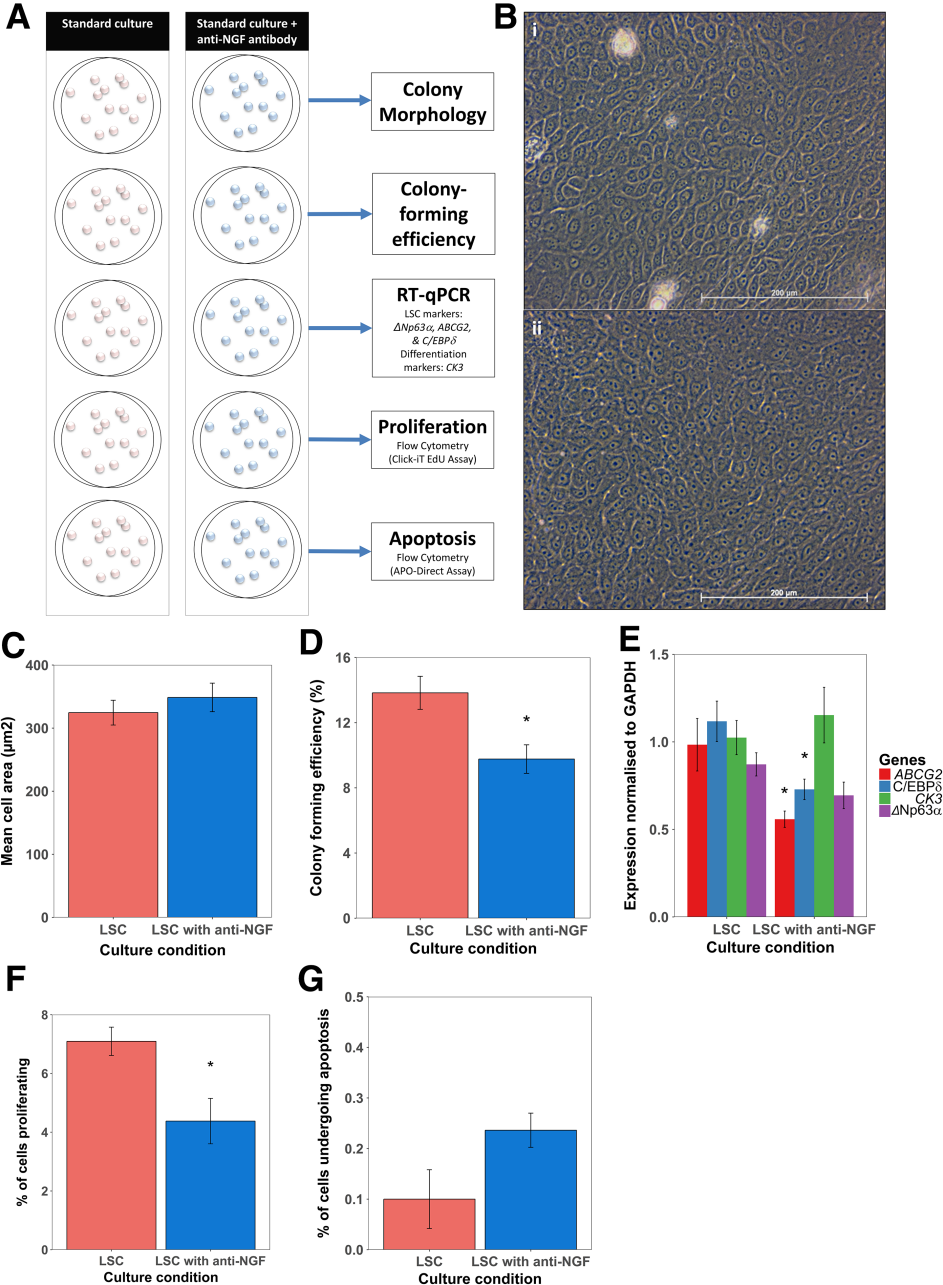
Nerve growth factor (NGF) blocking of limbal epithelial cultures decreases putative limbal stem cell (LSC) markers, colony‐forming efficiency (CFE), and proliferation. **(A):** Schema showing experimental set up for anti‐NGF studies. Each well shown was set up in biological triplicate. **(B):** Microphotographs of LSC culture at day 10 of culture in standard conditions (i) and in the presence of anti‐NGF (ii). Under a light microscope, the culture appearances are similar with tightly packed cells that are regular with large nuclei and scant cytoplasm. **(C):** There is no significant difference in mean cell area, as measured at day 10 of culture when LSCs are cultured in the presence of anti‐NGF (*p* = .43; *n* = 3; Student's *t* test; SEM). **(D):** CFE assay of standard culture versus standard culture with anti‐NGF at day 14 of culture. Culture in the presence of anti‐NGF results in a statistically significant reduction in CFE (*p* = .039; *n* = 3; Student's *t* test; SEM). **(E):** Bar charts showing RT‐qPCR results for the putative LSC markers *ABCG2*, *C/EBPδ*, Δ*Np63α*, and the corneal differentiation marker *CK3* at day 14 of culture. There is a statistically significant decrease in putative LSC markers *ABCG2* and *C/EBPδ* at day 14 of culture (*p* = .03 and .04, respectively; *n* = 3; Student's *t* test; SEM). There is a downward trend of Δ*Np63α* expression, and an upward trend of differentiation marker *CK3* when LSCs are cultured in the presence of anti‐NGF (*p* = .15 and .53, respectively; *n* = 3; Student's *t* test; SEM). **(F):** There is a statistically significant decrease in proliferation of LSCs cultured in the presence of anti‐NGF as measured at day 14 of culture (*p* = .042; *n* = 3; Student's *t* test; SEM). **(G):** There is no significant change in the levels of apoptosis when cells are cultured in the presence of anti‐NGF, as measured by APO‐DIRECT flow cytometry at day 14 of culture. There are very few apoptotic cells in both culture conditions, showing that the concentration of NGF was not causing direct toxicity to the cells (*p* = .12; *n* = 3; Student's *t* test; SEM).

To investigate the role of NGF signaling in LSCs, we blocked its function through the addition of a neutralizing anti‐NGF antibody throughout the culture period (for experimental design, please refer to Fig. 5A).

Microscopic examination of cells in standard culture and anti‐NGF conditions revealed tightly packed homogenous cells with a high nuclear:cytoplasm ratio (Fig. 5B (i) and (ii), respectively). There was no significant difference in cell area measured between these two conditions (*p* = .43; Fig. 5C).

LSCs cultured in the presence of anti‐NGF antibodies have a significantly reduced CFE compared to LSCs cultured under standard conditions (*p* = .039; Fig. 5D).

The expression of putative LSC markers *ABCG2*, *C/EBPδ*, and Δ*Np63α* and the corneal epithelial cell differentiation marker *CK3* mRNA was measured in LSCs cultured under both conditions by RT‐qPCR at day 14 of culture (Fig. 5E). The expression of *ABCG2* and *C/EBPδ* was significantly reduced in the anti‐NGF cell culture as compared to the standard culture (*p* = .03 and .04, respectively), whereas the expression of Δ*Np63α* also decreased, but this was not significant (*p* = .15). *CK3* expression was higher in the anti‐NGF cell culture, but this difference was not significant (*p* = .53). We reanalyzed the expression of *ABCG2*, *C/EBPδ*, Δ*Np63α*, and *CK3* mRNA at day 40 of culture in both standard and anti‐NGF conditions (Supporting Information Fig. S2B **)**. The expression of all three putative LSC markers, *ABCG2*, *C/EBPδ*, and Δ*Np63α* was significantly reduced in the anti‐NGF cell culture as compared to the standard culture (*p* = .0096, .0140, and .0312, respectively), whereas *CK3* expression was not significantly different between standard conditions and anti‐NGF conditions (*p* = .5236).

The fraction of the cell population undergoing mitosis in both culture conditions was inferred through measurement of the number of cells undergoing measurable DNA synthesis by the Click‐iT EdU assay and flow cytometry (Fig. 5F). We found that both culture conditions contained a cohort of cells actively undergoing DNA replication. Significantly fewer cells cultured in the presence of anti‐NGF were undergoing detectable DNA synthesis as compared to cells cultured under standard conditions (*p* = .042).

The fraction of the cell population undergoing apoptosis was inferred through the measurement of fluorescein‐labeled dUTP, which is incorporated into DNA at strand breaks in the APO‐DIRECT assay (Fig. 5G). We found no significant difference in the proportion of cells undergoing apoptosis in both culture conditions (*p* = .12). These cells comprised a small proportion of the cell population in both standard culture and anti‐NGF culture conditions.

### Addition of NGF to Culture Media Promotes Expression of LSCs Markers

**Figure 6:**
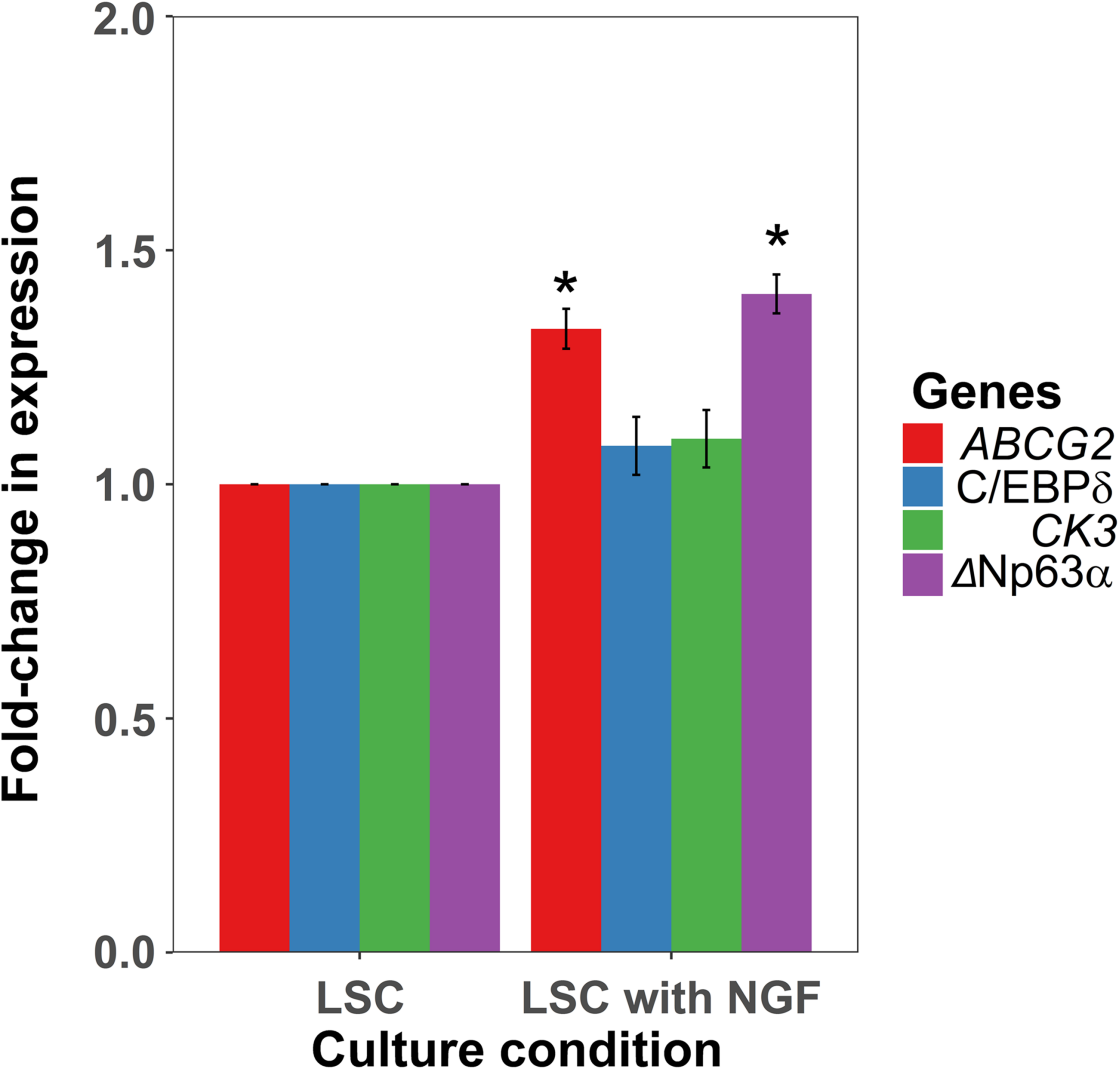
Nerve growth factor (NGF) addition to limbal epithelial cultures increases putative limbal stem cell (LSC) markers. Bar charts showing RT‐qPCR results for the putative LSC markers *ABCG2*, *C/EBPδ*, Δ*Np63α*, and the corneal differentiation marker *CK3* at day 40 of culture. There is a statistically significant increase in putative LSC markers Δ*Np63α* and *ABCG2* (*p* < 1 × 10^−7^ and *p* < 1 × 10^−6^, respectively; *n* = 3; Student's *t* test; SEM) whereas *CEPBδ* and *CK3* levels do not show significant differences between the groups (*p* = .099 and .104, respectively; *n* = 3; Student's *t* test; SEM).

We tested the hypothesis that NGF promotes the LSC phenotype by supplementing limbal culture media with NGF. The expression of putative markers of the LSC phenotype, *ABCG2*, *C/EBPδ*, and Δ*Np63α* and the corneal epithelial cell differentiation marker *CK3* were measured under control and NGF supplemented conditions (Fig. 6A). The expression of *ΔNp63α* and *ABCG2* increased significantly (*p* < 1 × 10^−7^ and *p* < 1 × 10^−6^, respectively), whereas the expression of *C/EBPδ* increased but only bordered on significant (*p* = .099). *CK3* expression did not significantly change between the control and NGF supplemented conditions (*p* = .104). Moreover, NGF addition to culture media prolonged proliferation of LSCs and maintained the CFE ability after the standard 40 days in culture. Namely, LSCs grown in NGF supplemented media were able to form colonies until passage 7, whereas those in standard culture conditions formed colonies until passage 5 (data not shown).

## Discussion

Stem cell populations are established in “niches”: anatomic locations that impose regulations on participation of stem cells in tissue regeneration, while supporting key stem cell characteristics. In LSCs, these characteristics may be summarized as: unlimited capacity for self‐renewal, slow‐cycling, and differentiation into corneal epithelium 1.

The LSC population is positioned to receive influences from a wide variety of cells which includes cell‐to‐cell contact, cell‐extracellular matrix contacts, and thirdly paracrine signaling factors and their receptors. Our previous work has shown that replication of the limbal niche in vitro results in directed differentiation of human ESC to corneal progenitors, thus suggesting a role for paracrine signaling in promoting the LSC phenotype 30.

It is already known that NGF and its receptors TrkA and p75^NTR^ are constitutively expressed in the basal limbus and cornea 18, 22, promote corneal epithelial proliferation and colony formation in vitro 21, 41, and corneal healing in vivo 27, 28, 29. Furthermore, expression of NGF and its high‐affinity receptor TrkA is higher in the limbus, the stem cell niche of LSCs, than in the corneal epithelium 17, 18, 19, 20, 21. As LSCs are known to replenish the corneal epithelium 2, 3, 4, 5, 6, 7, 8, it is likely that NGF and its receptors play a role in the regulation of the LSC niche. In this study, we found evidence to support this conclusion.

At the end of a long culture period (40 days) intended to induce LSC differentiation, limbal cultures displayed the morphological features of differentiated corneal epithelium, lost almost all capacity for colony formation, and expressed markers of differentiation. These results indicate differentiation of the limbal cultures to corneal epithelium during the culture period. Interestingly, p75^NTR^ underwent the greatest change in expression during differentiation, being downregulated 2.77‐fold. Analysis of enriched biological processes among these differentially regulated proteins showed that axon guidance was the most statistically significant and was clearly more significant than the rest of the top 20 biological processes. The cornea is amongst the most densely innervated tissues in the human body 42, and it therefore makes sense that a factor which stimulates corneal healing will also facilitate axon guidance to newly generated epithelium. Corneal epithelium and trigeminal neurons exist in a mutualistic relationship whereby neurons release paracrine factors which stimulate epithelial growth and differentiation, while corneal epithelial cells in turn release factors such as NGF which promote neurite extension and survival 20. It is already known that denervation impairs corneal healing, and that this impairment can be reversed by topical application of NGF 43. Among the differentially expressed proteins designated under the biological process term “axon guidance” were p75^NTR^ and its downstream mediator MAPK 44. MAPK/ERK signaling is known to promote corneal epithelial cell survival and migration in corneal healing 45, 46. Hong et al. have shown that MAPK/ERK signaling is activated after administration of NGF and activation of TrkA, in a dose‐dependent manner 47. They also showed that administration of NGF to primary human limbal cultures promotes G_1_‐S transition through activation of D‐cyclins. CDKs were amongst our proteins which made the cut‐off, with CDK6, p16, and cyclin D1 being downregulated on differentiation. Furthermore, negative regulation of the G_1_‐S transition also featured in the top 20 biological processes.

Analysis of signaling pathways which were differentially expressed between phenotypically stem cell and differentiated cultures showed that the cellular response to stress was the most significant pathway term, consistent with the role of LSC differentiation in response to corneal injury 19, 48. Functional network analysis of these enriched pathways shows that NGF signaling exists at the top of a hierarchical network, controlling cell cycle, Toll‐like receptor, senescence regulatory pathways, and VEGF signaling. The cornea is the eye's first line of defense against pathogens and therefore TLRs are likely to play an important role in the cornea's innate defense. NGF is already known to regulate corneal innate immunity. Micera et al. have shown that TLR4 is upregulated by NGF in a dose‐dependent fashion in primary cultures of vernal keratoconjunctivitis‐derived conjunctival epithelial cells 49. This suggests that after corneal injury, NGF may promote expression of TLR4 in anticipation of a potential infection. Interestingly, NGF has been shown to directly promote VEGF synthesis and release on the ocular surface, as indicated by our pathway analysis. Darci et al. used an in vivo corneal micropocket assay to delivery NGF in a mouse model of corneal healing and showed that NGF stimulates lymphangiogensis and VEGF expression. Blockade of VEGF signaling prevented NGF‐dependant lymphangiogenesis 12. Sornelli et al. used immunocytochemical analysis to show that NGF stimulates the synthesis and release of VEGF in human corneal endothelial cells in a dose‐dependent manner 50.

The NGF receptor p75^NTR^ is downregulated upon differentiation of LSCs, and as we have shown by Western blotting and RT‐qPCR, NGF is also downregulated. Pathways known to be involved in corneal healing have been differentially regulated in concert with NGF signaling and are plausibly regulated by NGF signaling. This is in keeping with the known pleiotropy of NGF in corneal epithelium and illustrates its importance as possibly a central paracrine factor in corneal healing, regulating processes as diverse as the cell motility and innate immunity. Interestingly, gene expression analysis shows a nonsignificant decrease in TrkA expression with LSC differentiation that is not as dramatic as with NGF and its nonspecific receptor p75^NTR^. On the other hand, no convincing decrease in TrkA expression with differentiation is shown by Western blot. This is despite TrkA being an NGF high‐affinity receptor having the same distribution in the limbus and cornea as NGF and having a known pro‐survival trophic role 18, 23. This discrepancy may be explained by examining the role of TrkA in concert with p75^NTR^, which is known to bind to and increase the affinity of TrkA for NGF as well as modulating its downstream actions 23, 51. Furthermore, when activated in isolation, p75^NTR^ produces pro‐apoptotic signals as opposed to the trophic and survival signals produced when activated by NGF in concert with TrkA 23, 24. Mutant mice that do not express the full‐length neurotrophin‐binding form of p75^NTR^ exhibit severe peripheral sensory deficits. Immunohistochemical analysis of the footpads of p75^NTR^ knockout mice showed markedly reduced sensory innervation and these mice developed peripheral ulcers 52. These findings together with our data point to a pro‐survival role of p75^NTR^ in concert with TrkA in limbal cell cultures. Presumably the downregulation of NGF and p75^NTR^ with the differentiation of LSCs reduces pro‐survival and trophic signaling by the NGF‐TrkA pathway. The pathways of NGF signaling in LSC cultures and the corneal epithelium should be the subject of further investigation.

Our results show that NGF and its receptor p75^NTR^ are highly expressed in LSCs and downregulated upon in vitro differentiation. Blocking of NGF signaling resulted in LSC differentiation as shown by a drop in CFE, decrease in expression of putative LSC markers *ABCG2* and *C/EBPδ* and decrease in cell proliferation. Thus, not only does NGF maintain the LSC phenotype, it is also a stimulator of LSC proliferation. This role in proliferation may be via the p75^NTR^ receptor that Qi et al. have noted is found in a distribution associated with transit amplifying cells 18. Interestingly, we found the anti‐NGF reduced expression of putative LSC markers (with no change in *CK3* expression) even after 40 days of in vitro culture, suggesting that at day 40 of culture these cells are not terminally differentiated and are cells in the transit amplifying state, a possibility which should be the subject of future investigation. Addition of rhNGF to LSC cultures resulted in increased expression of LSC markers Δ*Np63α* and *ABCG2*. This finding is further supported by prolonged exposure of LSC cultures to anti‐NGF which resulted in a significant decrease of all three putative LSC markers, *ABCG2*, *C/EBPδ*, and Δ*Np63α*. Significantly, supplementation of LSC culture medium with rhNGF extended the life span and colony forming ability of LSC cultures in vitro. Together, these results show that NGF is an ideal candidate for a paracrine stimulator of corneal healing and raises the possibility of its use in supporting the growth and survival of LSC transplants. Prolonged life span and colony‐forming capacity in vitro following addition of NGF to the culture medium is clinically significant since it could enable propagation of donor cells for a longer period of time and potentially increase the numbers of LSCs available for transplant. Furthermore, p75^NTR^ may be useful marker of the LSC phenotype in limbal cultures and an indicator of the suitability for transplant of in vitro expanded LSC cultures. Limbal biopsies are taken from the healthy sections of eye and expanded in vitro before transplantation as a treatment for LSCD 1.

The NGF content of patient tears is known to correlate with nerve regeneration following keratoplasty 48, topical application of NGF is known to promote surgical wound healing 29, and local overexpression in rats improves corneal graft survival 53. A randomized control trial currently in progress (NCT03035864) aims to study the safety and efficacy of topical application of recombinant human NGF in the treatment of ocular discomfort after surgery 54. In a recent phase II clinical trial examining the safety and efficacy of the use of topical rhNGF in the treatment of neurotrophic keratitis, 58% of those patients receiving 20 μg/ml rhNGF achieved corneal healing compared to 19.6% of vehicle‐treated patients 55. Thus, there are numerous potential therapeutic applications for NGF.

Furthermore, therapeutic application of NGF may improve graft survival after keratoplasty. We have now shown that NGF maintains the LSC phenotype and thus is likely to play a key role in regulation of the LSC niche. Given these findings, one may posit that topical application of NGF to patients with partial LSCD may augment the remaining LSCs and prevent need for LSC transplantation. Furthermore, understanding the factors of the LSC niche which promote maintenance of the LSC phenotype will aid the development of optimal in vitro cell‐free growth conditions for LSCs and will allow experimental design to more closely mimic that of the limbal microenvironment. Ultimately, this will aid the design of LSC‐based therapies.

## Author Contributions

S.K. and S.B.: experimental design, data acquisition and analysis, manuscript writing. A.E.G.: data analysis, manuscript writing. M.K.‐A.: data acquisition and analysis, contributed to manuscript writing. F.C.F.: study design, manuscript writing and fund raising. M.L.: study design, data acquisition and analysis, manuscript writing and fund raising. All authors approved the final version of the manuscript.

## Disclosure of Potential Conflicts of Interest

The authors indicated no potential conflicts of interest.

## Supporting information


**Figure S1: LSC morphology is observed at the beginning of culture and differentiated features are seen towards the end. Differentiation is associated with an increase in cell area.** (**A‐C**) Micro‐photographs of LSC‐3TC co‐culture taken with a light microscope at day 0 (**A**), day 7 (**B**) and day 40 (**C**). (**D‐G**) Micro‐photographs taken with a light microscope showing the changes in cell size and morphology during differentiation protocol at days 10, 20, 30 and 40 (**D‐G**, respectively). The cells become progressively larger throughout the culture process (**H**) (p < 1e‐16; n = 3; one‐way ANOVA; SEM). Between‐group analysis is shown in Table S4.

**Figure S2: Expression of the putative limbal stem cell markers *ABCG2, C/EBPδ, ΔNp63α* and the corneal differentiation marker *CK3* by RT‐qPCR at day 40 of culture.** (**A**) Western blot from Figure 4A with full gels and ladders shown. Red dotted boxes encapsulate the protein of interest. Shown are TrkA (**i, arrow**), the raw TrkA gel image to show the distinct bands (**ii, arrow**), p75^NTR^ (**iii, full‐length and short variants indicated by arrows and arrowheads, respectively**), NGF (**iv; mature and pro‐NGF indicated by arrows and arrowhead, respectively**) and total protein loading (**v**). (**B**) Gene expression analysis of LSCs cultured until day 40 under standard conditions or standard conditions plus anti‐NGF antibody. The expression of *ABCG2* was significantly reduced with NGF blocking (p = .0096), as well as the expression of *C/EBPδ* and *ΔNp63α* (p = .0140 and p = .0312, respectively). The expression of *CK3* was not significantly different between standard conditions and anti‐NGF conditions (p = .5236). Between‐group analysis is shown in Table S4.

**Table S1:** Primers used in the RT‐qPCR experiments.

**Table S2:** Details of the primary and secondary antibodies used in the Western blot.

**Table S3:** Differentially expressed proteins which made the cut‐off fold change of ≥1.2 (n = 109). FC (fold‐change), SE (standard error), SD (standard deviation).

**Table S4:** Between‐group analyses for each of the one‐way ANOVA tests carried out in this study. Tukey's Honest Significant Difference (HSD) was used for post‐hoc analysis.
