# Supplementary figures and images for: The Role of Nerve Growth Factor in Maintaining Proliferative Capacity, Colony‐Forming Efficiency, and the Limbal Stem Cell Phenotype
Source: Stem Cells. 2018 Dec 31;37(1):139–49. doi: 10.1002/stem.2921 (PMC6334532; doi:10.1002/stem.2921)

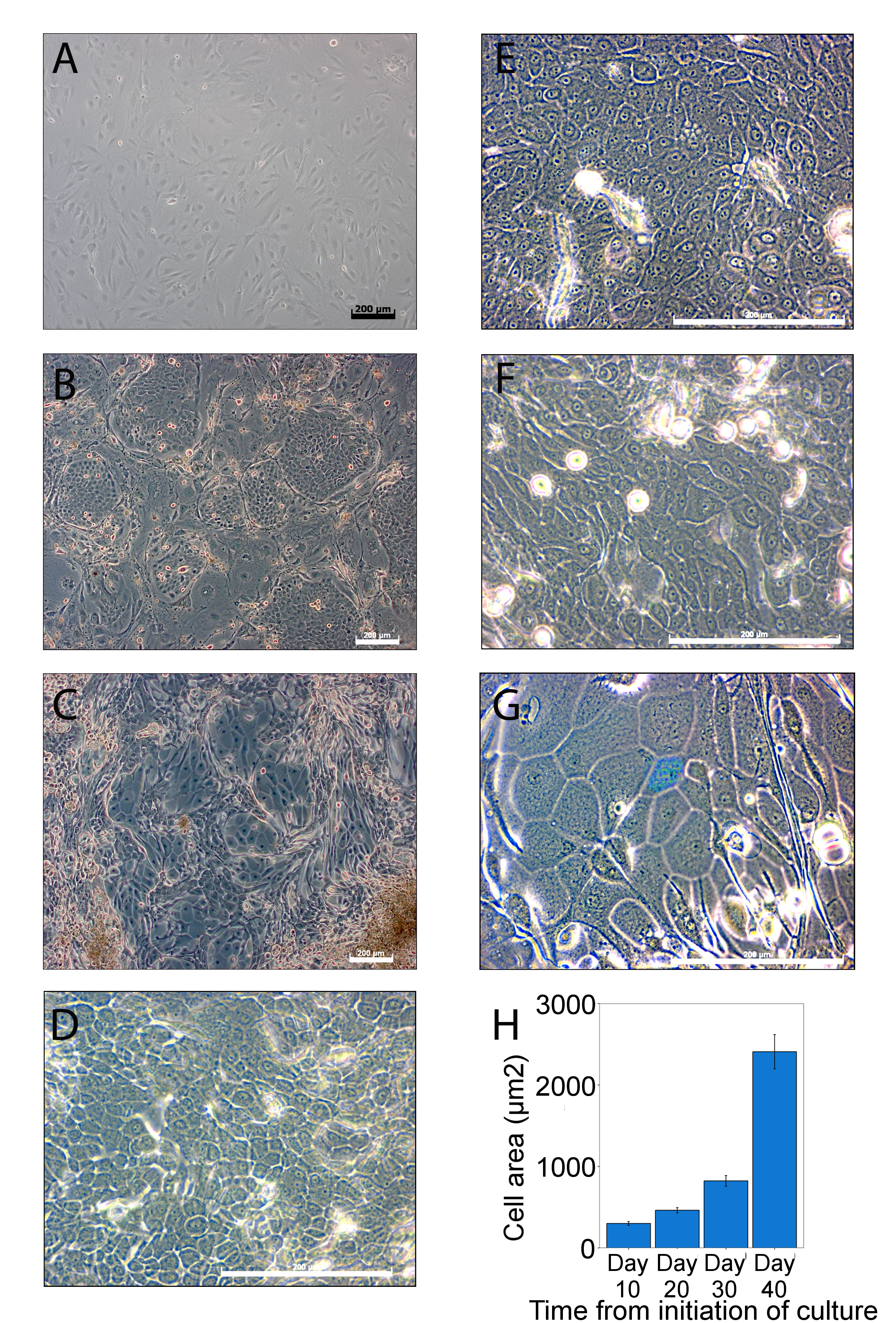

Supplement: Supplementary file 1 — Figure S1: LSC morphology is observed at the beginning of culture and differentiated features are seen towards the end. Differentiation is associated with an increase in cell area. (A‐C) Micro‐photographs of LSC‐3TC co‐culture taken with a light microscope at day 0 (A), day 7 (B) and day 40 (C). (D‐G) Micro‐photographs taken with a light microscope showing the changes in cell size and morphology during differentiation protocol at days 10, 20, 30 and 40 (D‐G, respectively). The cells become progressively larger throughout the culture process (H) (p < 1e‐16; n = 3; one‐way ANOVA; SEM). Between‐group analysis is shown in Table S4. [file STEM-37-139-s001.tiff]

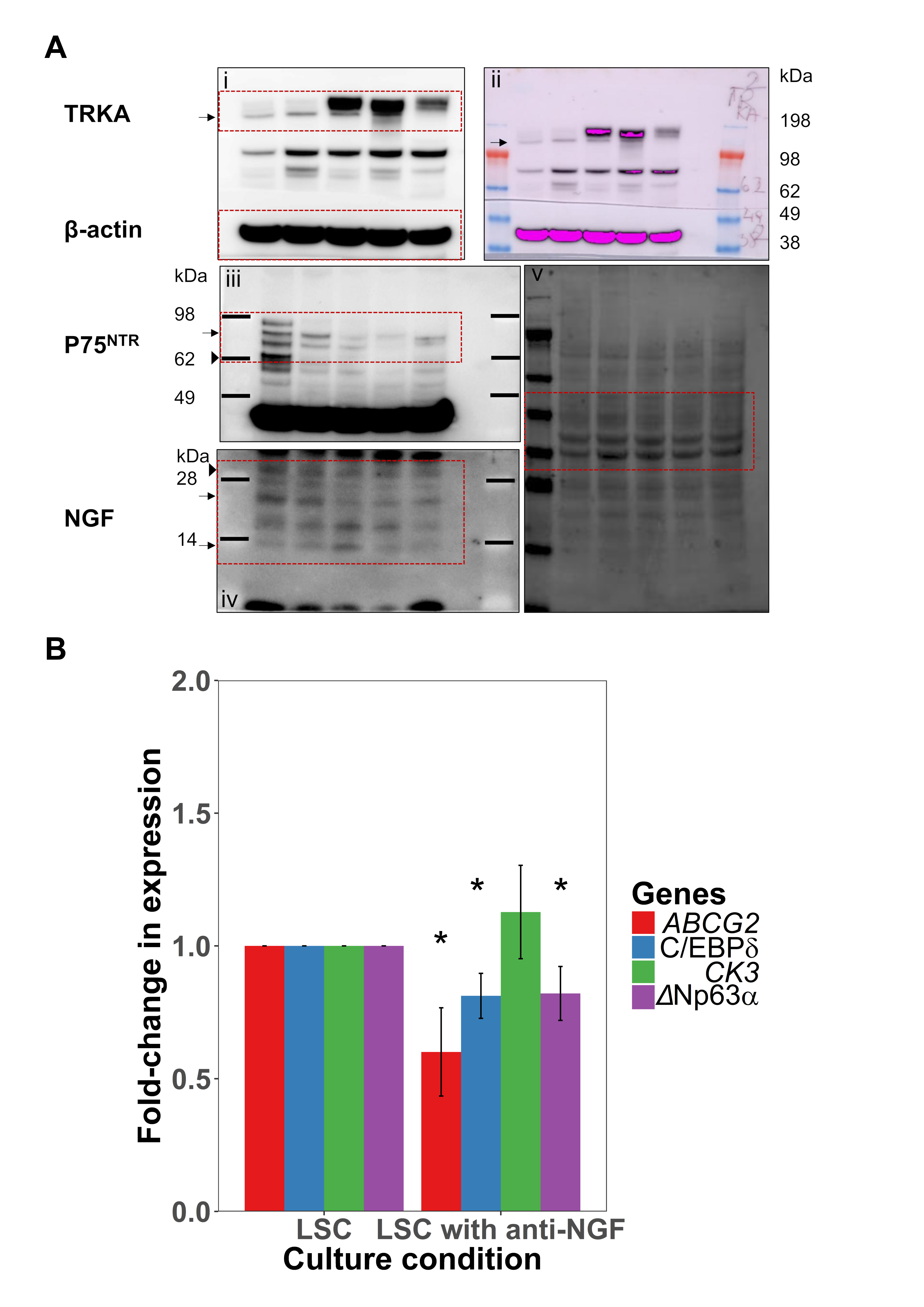

Supplement: Supplementary file 2 — Figure S2: Expression of the putative limbal stem cell markers ABCG2, C/EBPδ, ΔNp63α and the corneal differentiation marker CK3 by RT‐qPCR at day 40 of culture. (A) Western blot from Figure 4A with full gels and ladders shown. Red dotted boxes encapsulate the protein of interest. Shown are TrkA (i, arrow), the raw TrkA gel image to show the distinct bands (ii, arrow), p75NTR (iii, full‐length and short variants indicated by arrows and arrowheads, respectively), NGF (iv; mature and pro‐NGF indicated by arrows and arrowhead, respectively) and total protein loading (v). (B) Gene expression analysis of LSCs cultured until day 40 under standard conditions or standard conditions plus anti‐NGF antibody. The expression of ABCG2 was significantly reduced with NGF blocking (p = .0096), as well as the expression of C/EBPδ and ΔNp63α (p = .0140 and p = .0312, respectively). The expression of CK3 was not significantly different between standard conditions and anti‐NGF conditions (p = .5236). Between‐group analysis is shown in Table S4. [file STEM-37-139-s002.tif]
