# Supplementary material for: The Role of Nerve Growth Factor in Maintaining Proliferative Capacity, Colony‐Forming Efficiency, and the Limbal Stem Cell Phenotype
Source: Stem Cells. 2018 Dec 31;37(1):139–49. doi: 10.1002/stem.2921 (PMC6334532; doi:10.1002/stem.2921)
Supplement: Supplementary file 3 — Table S1: Primers used in the RT‐qPCR experiments. [file STEM-37-139-s003.docx]

**Table S1:** Primers used in the RT-qPCR experiments

| **Gene** | **Primer** | **Sequence** |  | |  |
| --- | --- | --- | --- | --- | --- |
| *GAPDH* | forward | TGCACCACCAACTGCTTAGC | |  | |
| *GAPDH* | reverse | GGCATGGACTGTGGTCATGAG | |  |  |
| *C/EBPδ* | forward | ACTTCAGCGCCTACATCGAC | |  | |
| *C/EBPδ* | reverse | GCCTTGTGATTGCTGTTGAA | |  |  |
| *δNp63α* | forward | GAAACGTACAGGCAACAGCA | |  | |
| *δΝp63α* | reverse | GCTGCTGAGGGTTGATAAGC | |  |  |
| *ABCG2* | forward | GCGACCTGCCAATTTCAAATG | |  | |
| *ABCG2* | reverse | GACCCTGTTAATCCGTTCGTTT | |  |  |
| *CK3* | forward | GGATGTGGACAGTGCCTATATG | |  | |
| *CK3* | reverse | AGATAGCTCAGCGTCGTAGAG | |  |  |
| *NGF* | forward | AGGGAGCAGCTTTCTATCCTG | |  | |
| *NGF* | reverse | GGCAGTGTCAAGGGAATGC | |  |  |
| *NGFR (p75)* | forward | CCTACGGCTACTACCAGGATG | |  | |
| *NGFR (p75)* | reverse | CACACGGTGTTCTGCTTGTC | |  |  |
| *TrkA* | forward | ATGGACAACCCTTTCGAGTTCAAC | |  | |
| *TrkA* | reverse | GACCCCAAAAGGTGTTTCGTCC | |  |  |
