# Supplementary material for: The Role of Nerve Growth Factor in Maintaining Proliferative Capacity, Colony‐Forming Efficiency, and the Limbal Stem Cell Phenotype
Source: Stem Cells. 2018 Dec 31;37(1):139–49. doi: 10.1002/stem.2921 (PMC6334532; doi:10.1002/stem.2921)
Supplement: Supplementary file 4 — Table S2: Details of the primary and secondary antibodies used in the Western blot. [file STEM-37-139-s004.docx]

**Table S2:** Details of the primary and secondary antibodies used in the Western blot

| Protein | Company | Product Code | Molecular weight (kDa) | Dilution | Source | Human detection? | Secondary reagent |
| --- | --- | --- | --- | --- | --- | --- | --- |
| NGF | Santa Cruz  Biotechnology  Inc. | NGF (E-12): sc-365944 | 13 (mature) 27 (precursor) | 1:100 | Mouse | Yes | Polyclonal rabbit anti-mouse immunoglobulins HRP Dako at  1:2000 |
| TrkA | Abcam | Ab76291 | 145 | 1:500 | Rabbit | Yes | Polyclonal swine anti-rabbit immunoglobulins HRP Dako at 1:2000 |
| p75^NTR^ | Cell Signalling | D8A8  #4201 | 75 | 1:500 | Rabbit | Yes | Polyclonal swine anti-rabbit immunoglobulins HRP Dako at 1:2000 |
| Β-actin | Santa Cruz  Biotechnology  Inc. | β-actin(C4):  sc-47778 | 43 | 1:40,000 | Mouse | Yes | Polyclonal rabbit anti-mouse immunoglobulins HRP Dako at 1:2000 |
