# Supplementary material for: The Role of Nerve Growth Factor in Maintaining Proliferative Capacity, Colony‐Forming Efficiency, and the Limbal Stem Cell Phenotype
Source: Stem Cells. 2018 Dec 31;37(1):139–49. doi: 10.1002/stem.2921 (PMC6334532; doi:10.1002/stem.2921)
Supplement: Supplementary file 5 — Table S3: Differentially expressed proteins which made the cut‐off fold change of ≥1.2 (n = 109). FC (fold‐change), SE (standard error), SD (standard deviation). [file STEM-37-139-s005.docx]

**Table S3:** Differentially expressed proteins which made the cut-off fold change of ≥1.2 (n=109). FC (fold-change), SE (standard error), SD (standard deviation). ^†^The upregulated Neurofilament 200 is expression as detected by a monoclonal antibody, while the downregulated neurofilament 200 is detected via a polyclonal antibody.

| **protein** | **FC** | **SE** | **SD** | **p-value** |
| --- | --- | --- | --- | --- |
| Nerve growth factor receptor | 2.771 | 0.367 | 0.518 | 0.081 |
| cytokeratin 4 | 2.124 | 0.089 | 0.126 | 0.035 |
| Tryptophane Hydroxlase | 2.108 | 0.066 | 0.093 | 0.027 |
| Internexin | 2.105 | 0.443 | 0.627 | 0.172 |
| GRP1(ARNO3) | 2.094 | 0.031 | 0.044 | 0.013 |
| Cdc7 Kinase | 2.002 | 0.028 | 0.040 | 0.013 |
| Neurofilament 200 – monoclonal ab† | 1.947 | 0.182 | 0.258 | 0.088 |
| Tyrosin Hydroxlase | 1.925 | 0.137 | 0.194 | 0.069 |
| ARNO | 1.881 | 0.007 | 0.009 | 0.004 |
| cytokeratin 8.13 | 1.878 | 0.077 | 0.108 | 0.041 |
| CNPase | 1.845 | 0.287 | 0.406 | 0.157 |
| AP-1/cJUN | 1.841 | 0.102 | 0.145 | 0.058 |
| Dystrophin | 1.838 | 0.110 | 0.156 | 0.062 |
| EGF Receptor | 1.821 | 0.115 | 0.162 | 0.067 |
| MAPK non phosporylated | 1.792 | 0.059 | 0.083 | 0.036 |
| NFkB | 1.784 | 0.080 | 0.113 | 0.049 |
| NAK | 1.709 | 0.018 | 0.025 | 0.012 |
| PKB/AKT (mono) | 1.675 | 0.349 | 0.494 | 0.240 |
| Topoisomerase 1 | 1.657 | 0.001 | 0.001 | 0.001 |
| MAP1 | 1.617 | 0.032 | 0.046 | 0.026 |
| bTubulin IV | 1.587 | 0.234 | 0.468 | 0.049 |
| NTF2 | 1.578 | 0.085 | 0.120 | 0.075 |
| ARTS | 1.575 | 0.032 | 0.045 | 0.029 |
| cytokeratin 8.60 | 1.574 | 0.091 | 0.129 | 0.080 |
| cytokeratin 19 | 1.541 | 0.115 | 0.163 | 0.109 |
| SUV39H1 | 1.513 | 0.014 | 0.020 | 0.015 |
| cdk6 | 1.489 | 0.001 | 0.002 | 0.001 |
| Ezrin | 1.488 | 0.379 | 0.535 | 0.353 |
| HSP90 | 1.478 | 0.057 | 0.081 | 0.063 |
| Dystrophin | 1.473 | 0.007 | 0.010 | 0.008 |
| cytokeratin 13 | 1.465 | 0.051 | 0.073 | 0.058 |
| PKC g (mono) | 1.463 | 0.017 | 0.023 | 0.019 |
| cytokeratin 7 | 1.460 | 0.023 | 0.033 | 0.027 |
| b-NOS (mono) | 1.449 | 0.083 | 0.118 | 0.098 |
| PAK-pSer212 | 1.448 | 0.067 | 0.095 | 0.079 |
| b-Actin mono. | 1.442 | 0.043 | 0.061 | 0.052 |
| cytokeratin 8.12 | 1.426 | 0.112 | 0.159 | 0.139 |
| i-NOS (mono) | 1.410 | 0.183 | 0.259 | 0.228 |
| DAPK pSer308 | 1.392 | 0.155 | 0.219 | 0.206 |
| MAP2 | 1.391 | 0.197 | 0.278 | 0.255 |
| GAP1 | 1.388 | 0.013 | 0.019 | 0.019 |
| Actin mono. | 1.383 | 0.126 | 0.178 | 0.173 |
| DOPA Decarboxylase | 1.375 | 0.184 | 0.260 | 0.251 |
| hnRNP M3-M4 | 1.373 | 0.002 | 0.003 | 0.003 |
| p16 | 1.370 | 0.000 | 0.001 | 0.001 |
| Cystatin A | 1.367 | 0.014 | 0.020 | 0.021 |
| Caspase8 | 1.351 | 0.086 | 0.121 | 0.132 |
| Caspase 10 | 1.330 | 0.185 | 0.262 | 0.287 |
| Adaptin | 1.327 | 0.028 | 0.040 | 0.047 |
| FAK-pSer772 | 1.325 | 0.013 | 0.019 | 0.022 |
| p38 MAPK activated | 1.322 | 0.012 | 0.016 | 0.020 |
| S-100 b | 1.319 | 0.140 | 0.198 | 0.232 |
| Cyclin A (poly) | 1.298 | 0.068 | 0.096 | 0.126 |
| Phospolipase c g 1 | 1.288 | 0.019 | 0.027 | 0.038 |
| bTubulin polyglutamylated | 1.281 | 0.037 | 0.074 | 0.003 |
| Aop-1 | 1.279 | 0.060 | 0.085 | 0.120 |
| Cyclin D1 | 1.271 | 0.064 | 0.091 | 0.132 |
| cdk4 | 1.267 | 0.047 | 0.066 | 0.098 |
| Smad4 | 1.262 | 0.171 | 0.241 | 0.332 |
| pan Cytokeratin | 1.260 | 0.070 | 0.098 | 0.149 |
| Desmin | 1.240 | 0.490 | 0.693 | 0.664 |
| Tau-pSer199/202 | 1.238 | 0.000 | 0.001 | 0.001 |
| Pyk2 - pTyr580 | 1.233 | 0.102 | 0.145 | 0.240 |
| c-Abl | 1.228 | 0.033 | 0.046 | 0.082 |
| Cyclin D3 | 1.204 | 0.072 | 0.101 | 0.197 |
| pHistone H3-pSer28 | 0.801 | 0.035 | 0.050 | 0.125 |
| Actopaxin | 0.796 | 0.050 | 0.070 | 0.170 |
| PTEN (mono) | 0.796 | 0.014 | 0.019 | 0.048 |
| Glutamine Syntethase | 0.785 | 0.008 | 0.012 | 0.028 |
| E2F1 | 0.784 | 0.009 | 0.013 | 0.030 |
| pHistone H3-pSer10 | 0.783 | 0.010 | 0.014 | 0.032 |
| Cyclin B1 | 0.782 | 0.009 | 0.013 | 0.029 |
| Caspase 10 | 0.781 | 0.003 | 0.005 | 0.011 |
| Synuclein | 0.778 | 0.016 | 0.023 | 0.054 |
| Acetyl Histone 3-Ac-Lys 9 | 0.760 | 0.049 | 0.069 | 0.146 |
| aCatenin | 0.760 | 0.044 | 0.062 | 0.131 |
| Actin | 0.757 | 0.040 | 0.056 | 0.119 |
| Grb-2 | 0.751 | 0.023 | 0.032 | 0.066 |
| Amyloid Precursor Protein (APP) | 0.749 | 0.019 | 0.027 | 0.057 |
| Caspase 3 | 0.749 | 0.018 | 0.025 | 0.052 |
| Glutamate receptor NMDAR 2a | 0.747 | 0.039 | 0.055 | 0.112 |
| Glutamic Acid Decarboxlase (GAD65/67) | 0.747 | 0.026 | 0.037 | 0.076 |
| RAN | 0.730 | 0.004 | 0.006 | 0.012 |
| ERK5 | 0.727 | 0.033 | 0.047 | 0.091 |
| Caveolin1 | 0.726 | 0.033 | 0.046 | 0.089 |
| Connexin 32 | 0.723 | 0.026 | 0.037 | 0.072 |
| Calcineurin | 0.708 | 0.015 | 0.021 | 0.038 |
| Estrogen Receptor | 0.705 | 0.006 | 0.008 | 0.015 |
| PKB/AKT | 0.684 | 0.003 | 0.004 | 0.007 |
| p19 | 0.676 | 0.049 | 0.069 | 0.116 |
| p38 MAPK | 0.676 | 0.022 | 0.032 | 0.053 |
| chk1 | 0.673 | 0.008 | 0.012 | 0.020 |
| HDAC1 | 0.664 | 0.012 | 0.016 | 0.027 |
| HDAC2 | 0.651 | 0.015 | 0.021 | 0.033 |
| Neurofilament 200 – polyclonal ab† | 0.647 | 0.015 | 0.022 | 0.035 |
| Caspase 3 active | 0.643 | 0.003 | 0.004 | 0.006 |
| Cofilin | 0.642 | 0.010 | 0.015 | 0.023 |
| Nedd8 | 0.634 | 0.001 | 0.001 | 0.002 |
| Calponin | 0.632 | 0.033 | 0.047 | 0.072 |
| PAR4 | 0.623 | 0.013 | 0.018 | 0.027 |
| p35 | 0.621 | 0.026 | 0.036 | 0.055 |
| Acetyl and Phospho Histone 3 | 0.616 | 0.005 | 0.007 | 0.011 |
| e-NOS | 0.609 | 0.018 | 0.025 | 0.038 |
| MAP Kinase (Erk1) | 0.599 | 0.016 | 0.023 | 0.034 |
| SMAC/DIABLO | 0.591 | 0.011 | 0.015 | 0.022 |
| e-NOS | 0.586 | 0.014 | 0.019 | 0.027 |
| MAPK activated protein kinase-2 | 0.570 | 0.007 | 0.009 | 0.013 |
| bCOP | 0.558 | 0.023 | 0.032 | 0.044 |
| cdc27 | 0.536 | 0.011 | 0.016 | 0.021 |
