# Supplementary material for: The Role of Nerve Growth Factor in Maintaining Proliferative Capacity, Colony‐Forming Efficiency, and the Limbal Stem Cell Phenotype
Source: Stem Cells. 2018 Dec 31;37(1):139–49. doi: 10.1002/stem.2921 (PMC6334532; doi:10.1002/stem.2921)
Supplement: Supplementary file 6 — Table S4: Between‐group analyses for each of the one‐way ANOVA tests carried out in this study. Tukey's Honest Significant Difference (HSD) was used for post‐hoc analysis. [file STEM-37-139-s006.docx]

**Table S4:** Between-group analyses for each of the one-way ANOVA tests carried out in this study. Tukey’s Honest Significant Difference (HSD) was used for post-hoc analysis.

| Multiple Comparisons - FIG2A | | | | | | | |  | |  | | |  | | |  |
| --- | --- | --- | --- | --- | --- | --- | --- | --- | --- | --- | --- | --- | --- | --- | --- | --- |
| Dependent Variable: CFE | | | | | | | |  | |  | | |  | | |  |
| Tukey HSD | | | | |  | | |  | |  | | |  | | |  |
| (I) Day | | (J) Day | | Mean Difference (I-J) | | | Std. Error | | | | Sig. | | | 95% Confidence Interval | | |
|  |  | |  | | |  | | |  | | | Lower Bound | | | Upper Bound | |
| 10 | 20 | | 7.667* | | | 1.986 | | | 0.02 | | | 1.31 | | | 14.03 | |
|  | 30 | | 10.667* | | | 1.986 | | | 0.003 | | | 4.31 | | | 17.03 | |
|  | 40 | | 14.000* | | | 1.986 | | | <0.0001 | | | 7.64 | | | 20.36 | |
| 20 | 10 | | -7.667* | | | 1.986 | | | 0.02 | | | -14.03 | | | -1.31 | |
|  | 30 | | 3 | | | 1.986 | | | 0.475 | | | -3.36 | | | 9.36 | |
|  | 40 | | 6.333 | | | 1.986 | | | 0.051 | | | -0.03 | | | 12.69 | |
| 30 | 10 | | -10.667* | | | 1.986 | | | 0.003 | | | -17.03 | | | -4.31 | |
|  | 20 | | -3 | | | 1.986 | | | 0.475 | | | -9.36 | | | 3.36 | |
|  | 40 | | 3.333 | | | 1.986 | | | 0.393 | | | -3.03 | | | 9.69 | |
| 40 | 10 | | -14.000* | | | 1.986 | | | <0.0001 | | | -20.36 | | | -7.64 | |
|  | 20 | | -6.333 | | | 1.986 | | | 0.051 | | | -12.69 | | | 0.03 | |
|  | 30 | | -3.333 | | | 1.986 | | | 0.393 | | | -9.69 | | | 3.03 | |
| * The mean difference is significant at the 0.05 level. | | | | | | | | | | | | | | | |  |

| Multiple Comparisons - ABCG2 (FIG2B) | | | |  | |  | | | |  | | |
| --- | --- | --- | --- | --- | --- | --- | --- | --- | --- | --- | --- | --- |
| Dependent Variable: Expression of ABCG2 | | |  | | | | |  | | | | |
| Tukey HSD | |  |  | | |  | |  | | |  | |
| (I) day | | (J) day | Mean Difference (I-J) | | | Std. Error | | Sig. | | | 95% Confidence Interval | |
|  |  | |  | |  | |  | | Lower Bound | | | Upper Bound |
| 10 | 20 | | -3.617541000* | | 0.719059 | | 0.006 | | -5.99774 | | | -1.23734 |
|  | 30 | | -7.215967500* | | 0.803933 | | <0.0001 | | -9.87711 | | | -4.55482 |
|  | 40 | | -5.590548000* | | 0.719059 | | <0.0001 | | -7.97075 | | | -3.21035 |
| 20 | 10 | | 3.617541000* | | 0.719059 | | 0.006 | | 1.237339 | | | 5.997743 |
|  | 30 | | -3.598426500* | | 0.803933 | | 0.012 | | -6.25957 | | | -0.93728 |
|  | 40 | | -1.97301 | | 0.719059 | | 0.105 | | -4.35321 | | | 0.407195 |
| 30 | 10 | | 7.215967500* | | 0.803933 | | <0.0001 | | 4.55482 | | | 9.877115 |
|  | 20 | | 3.598426500* | | 0.803933 | | 0.012 | | 0.937279 | | | 6.259574 |
|  | 40 | | 1.62542 | | 0.803933 | | 0.266 | | -1.03573 | | | 4.286567 |
| 40 | 10 | | 5.590548000* | | 0.719059 | | <0.0001 | | 3.210346 | | | 7.97075 |
|  | 20 | | 1.973007 | | 0.719059 | | 0.105 | | -0.4072 | | | 4.353209 |
|  | 30 | | -1.62542 | | 0.803933 | | 0.266 | | -4.28657 | | | 1.035728 |
| * The mean difference is significant at the 0.05 level. | | | | | |  | | | | | | |

| Multiple Comparisons - C/EBPδ (FIG2B) | | | | |  | |  | | | |  | | |
| --- | --- | --- | --- | --- | --- | --- | --- | --- | --- | --- | --- | --- | --- |
| Dependent Variable: Expression of C/EBPδ | | | |  | | | | |  | | | | |
| Tukey HSD | |  | |  | | |  | |  | | |  | |
| (I) day | | (J) day | | Mean Difference (I-J) | | | Std. Error | | Sig. | | | 95% Confidence Interval | |
|  |  | |  | | |  | |  | | Lower Bound | | | Upper Bound |
| 10 | 20 | | -2.634664500* | | | 0.236622 | | <0.0001 | | -3.41792 | | | -1.85141 |
|  | 30 | | -5.737253333* | | | 0.211641 | | <0.0001 | | -6.43782 | | | -5.03669 |
|  | 40 | | -5.661880667* | | | 0.211641 | | <0.0001 | | -6.36245 | | | -4.96131 |
| 20 | 10 | | 2.634664500* | | | 0.236622 | | <0.0001 | | 1.851408 | | | 3.417921 |
|  | 30 | | -3.102588833* | | | 0.236622 | | <0.0001 | | -3.88585 | | | -2.31933 |
|  | 40 | | -3.027216167* | | | 0.236622 | | <0.0001 | | -3.81047 | | | -2.24396 |
| 30 | 10 | | 5.737253333* | | | 0.211641 | | <0.0001 | | 5.036687 | | | 6.437819 |
|  | 20 | | 3.102588833* | | | 0.236622 | | <0.0001 | | 2.319332 | | | 3.885846 |
|  | 40 | | 0.075373 | | | 0.211641 | | 0.983 | | -0.62519 | | | 0.775939 |
| 40 | 10 | | 5.661880667* | | | 0.211641 | | <0.0001 | | 4.961315 | | | 6.362447 |
|  | 20 | | 3.027216167* | | | 0.236622 | | <0.0001 | | 2.243959 | | | 3.810473 |
|  | 30 | | -0.07537 | | | 0.211641 | | 0.983 | | -0.77594 | | | 0.625193 |
| * The mean difference is significant at the 0.05 level. | | | | | | |  | | | | | | |

| Multiple Comparisons - CK3 (FIG2B) | | | |  | | |  | | | |  | | | | |  |
| --- | --- | --- | --- | --- | --- | --- | --- | --- | --- | --- | --- | --- | --- | --- | --- | --- |
| Dependent Variable: Expression of CK3 | | | |  | | |  | | | |  | | | | |  |
| Tukey HSD | | |  | |  |  | | |  | | |  | |  | | |
| (I) day | | | (J) days | |  | Mean Difference (I-J) | | | Std. Error | | | Sig. | | 95% Confidence Interval | | |
|  |  | |  | |  | | |  | |  | | | Lower Bound | | Upper Bound | |
| 10 |  | | 20 | | 1.334583 | | | 0.538912 | | 0.149 | | | -0.4493 | | 3.11847 | |
|  |  | | 30 | | 3.45388667* | | | 0.602522 | | 0.003 | | | 1.459441 | | 5.448332 | |
|  |  | | 40 | | 4.57865667* | | | 0.538912 | | <0.0001 | | | 2.79477 | | 6.362543 | |
| 20 |  | | 10 | | -1.33458 | | | 0.538912 | | 0.149 | | | -3.11847 | | 0.449303 | |
|  |  | | 30 | | 2.11930333* | | | 0.602522 | | 0.038 | | | 0.124858 | | 4.113749 | |
|  |  | | 40 | | 3.24407333* | | | 0.538912 | | 0.002 | | | 1.460187 | | 5.02796 | |
| 30 |  | | 10 | | -3.45388667* | | | 0.602522 | | 0.003 | | | -5.44833 | | -1.45944 | |
|  |  | | 20 | | -2.11930333* | | | 0.602522 | | 0.038 | | | -4.11375 | | -0.12486 | |
|  |  | | 40 | | 1.12477 | | | 0.602522 | | 0.321 | | | -0.86968 | | 3.119216 | |
| 40 |  | | 10 | | -4.57865667* | | | 0.538912 | | <0.0001 | | | -6.36254 | | -2.79477 | |
|  |  | | 20 | | -3.24407333* | | | 0.538912 | | 0.002 | | | -5.02796 | | -1.46019 | |
|  |  | | 30 | | -1.12477 | | | 0.602522 | | 0.321 | | | -3.11922 | | 0.869676 | |
|  | | * The mean difference is significant at the 0.05 level. | | | | | | |  | | | | | | | |

| Multiple Comparisons - δNp63α (FIG2B) | | | | |  | |  | | | |  | | |
| --- | --- | --- | --- | --- | --- | --- | --- | --- | --- | --- | --- | --- | --- |
| Dependent Variable: Expression of δNp63α | | | |  | | | | |  | | | | |
| Tukey HSD | |  | |  | | |  | |  | | |  | |
| (I) day | | (J) day | | Mean Difference (I-J) | | | Std. Error | | Sig. | | | 95% Confidence Interval | |
|  |  | |  | | |  | |  | | Lower Bound | | | Upper Bound |
| 10 | 20 | | -4.906421000* | | | 0.260244 | | <0.0001 | | -5.73981 | | | -4.07303 |
|  | 30 | | -3.135727667* | | | 0.260244 | | <0.0001 | | -3.96912 | | | -2.30234 |
|  | 40 | | -5.315437667* | | | 0.260244 | | <0.0001 | | -6.14883 | | | -4.48205 |
| 20 | 10 | | 4.906421000* | | | 0.260244 | | <0.0001 | | 4.073029 | | | 5.739813 |
|  | 30 | | 1.770693333* | | | 0.260244 | | 0.001 | | 0.937302 | | | 2.604085 |
|  | 40 | | -0.40902 | | | 0.260244 | | 0.444 | | -1.24241 | | | 0.424375 |
| 30 | 10 | | 3.135727667* | | | 0.260244 | | <0.0001 | | 2.302336 | | | 3.969119 |
|  | 20 | | -1.770693333* | | | 0.260244 | | 0.001 | | -2.60409 | | | -0.9373 |
|  | 40 | | -2.179710000* | | | 0.260244 | | <0.0001 | | -3.0131 | | | -1.34632 |
| 40 | 10 | | 5.315437667* | | | 0.260244 | | <0.0001 | | 4.482046 | | | 6.148829 |
|  | 20 | | 0.409017 | | | 0.260244 | | 0.444 | | -0.42438 | | | 1.242408 |
|  | 30 | | 2.179710000* | | | 0.260244 | | <0.0001 | | 1.346318 | | | 3.013102 |
| * The mean difference is significant at the 0.05 level. | | | | | | |  | | | | | | |

| Multiple Comparisons - NGF (FIG4B) | | | | |  | |  | | | |  | | |
| --- | --- | --- | --- | --- | --- | --- | --- | --- | --- | --- | --- | --- | --- |
| Dependent Variable: expression | | | | |  | |  | | | |  | | |
| Tukey HSD | |  |  | | | |  | |  | | |  | |
| (I) day | | (J) day | Mean Difference (I-J) | | | | Std. Error | | Sig. | | | 95% Confidence Interval | |
|  |  | | |  | |  | |  | | Lower Bound | | | Upper Bound |
| 10 | 20 | | | .776345138333* | | 0.026147 | | <0.0001 | | 0.692612 | | | 0.860078 |
|  | 30 | | | .946368993333* | | 0.026147 | | <0.0001 | | 0.862636 | | | 1.030102 |
|  | 40 | | | .952218935333* | | 0.026147 | | <0.0001 | | 0.868486 | | | 1.035952 |
| 20 | 10 | | | -.776345138333* | | 0.026147 | | <0.0001 | | -0.86008 | | | -0.69261 |
|  | 30 | | | .170023855000* | | 0.026147 | | 0.001 | | 0.086291 | | | 0.253757 |
|  | 40 | | | .175873797000* | | 0.026147 | | 0.001 | | 0.092141 | | | 0.259607 |
| 30 | 10 | | | -.946368993333* | | 0.026147 | | <0.0001 | | -1.0301 | | | -0.86264 |
|  | 20 | | | -.170023855000* | | 0.026147 | | 0.001 | | -0.25376 | | | -0.08629 |
|  | 40 | | | 0.00585 | | 0.026147 | | 0.996 | | -0.07788 | | | 0.089583 |
| 40 | 10 | | | -.952218935333* | | 0.026147 | | <0.0001 | | -1.03595 | | | -0.86849 |
|  | 20 | | | -.175873797000* | | 0.026147 | | 0.001 | | -0.25961 | | | -0.09214 |
|  | 30 | | | -0.00585 | | 0.026147 | | 0.996 | | -0.08958 | | | 0.077883 |
| * The mean difference is significant at the 0.05 level. | | | | | | |  | | | | | | |

| Multiple Comparisons - p75NTR (FIG4B) | | | |  | | |  | | | | |  | | | |  |  |  |
| --- | --- | --- | --- | --- | --- | --- | --- | --- | --- | --- | --- | --- | --- | --- | --- | --- | --- | --- |
| Dependent Variable: expression | | | |  | | |  | | | | |  | | | |  |  |  |
| Tukey HSD | |  | | |  |  | | |  | | | |  | |  | | | |
| (I) day | | (J) day | | |  | Mean Difference (I-J) | | | Std. Error | | | | Sig. | | 95% Confidence Interval | | | |
|  |  |  | | |  | | |  | | |  | | | Lower Bound | | | Upper Bound |  |
| 10 |  | 20 | | | .346202539000* | | | 0.067482 | | | 0.004 | | | 0.130101 | | | 0.562304 |  |
|  |  | 30 | | | .659327783000* | | | 0.067482 | | | <0.0001 | | | 0.443227 | | | 0.875429 |  |
|  |  | 40 | | | .969184713667* | | | 0.067482 | | | <0.0001 | | | 0.753084 | | | 1.185286 |  |
| 20 |  | 10 | | | -.346202539000* | | | 0.067482 | | | 0.004 | | | -0.5623 | | | -0.1301 |  |
|  |  | 30 | | | .313125244000* | | | 0.067482 | | | 0.007 | | | 0.097024 | | | 0.529226 |  |
|  |  | 40 | | | .622982174667* | | | 0.067482 | | | <0.0001 | | | 0.406881 | | | 0.839083 |  |
| 30 |  | 10 | | | -.659327783000* | | | 0.067482 | | | <0.0001 | | | -0.87543 | | | -0.44323 |  |
|  |  | 20 | | | -.313125244000* | | | 0.067482 | | | 0.007 | | | -0.52923 | | | -0.09702 |  |
|  |  | 40 | | | .309856930667* | | | 0.067482 | | | 0.008 | | | 0.093756 | | | 0.525958 |  |
| 40 |  | 10 | | | -.969184713667* | | | 0.067482 | | | <0.0001 | | | -1.18529 | | | -0.75308 |  |
|  |  | 20 | | | -.622982174667* | | | 0.067482 | | | <0.0001 | | | -0.83908 | | | -0.40688 |  |
|  |  | 30 | | | -.309856930667* | | | 0.067482 | | | 0.008 | | | -0.52596 | | | -0.09376 |  |
|  | | | * The mean difference is significant at the 0.05 level. | | | | | | |  | | | | | | | |  |

| Multiple Comparisons - TrkA (FIG4B) | | |  | | | |  | | | |  | | |
| --- | --- | --- | --- | --- | --- | --- | --- | --- | --- | --- | --- | --- | --- |
| Dependent Variable: expression | | |  | | | |  | | | |  | | |
| Tukey HSD | |  | | |  | |  | |  | | |  | |
| (I) day | | (J) day | | | Mean Difference (I-J) | | Std. Error | | Sig. | | | 95% Confidence Interval | |
|  |  | | |  | |  | |  | | Lower Bound | | | Upper Bound |
| 10 | 20 | | | 0.192938 | | 0.15724 | | 0.628 | | -0.3106 | | | 0.696477 |
|  | 30 | | | 0.316895 | | 0.15724 | | 0.259 | | -0.18664 | | | 0.820434 |
|  | 40 | | | 0.369643 | | 0.15724 | | 0.165 | | -0.1339 | | | 0.873182 |
| 20 | 10 | | | -0.19294 | | 0.15724 | | 0.628 | | -0.69648 | | | 0.3106 |
|  | 30 | | | 0.123957 | | 0.15724 | | 0.858 | | -0.37958 | | | 0.627496 |
|  | 40 | | | 0.176705 | | 0.15724 | | 0.686 | | -0.32683 | | | 0.680244 |
| 30 | 10 | | | -0.3169 | | 0.15724 | | 0.259 | | -0.82043 | | | 0.186643 |
|  | 20 | | | -0.12396 | | 0.15724 | | 0.858 | | -0.6275 | | | 0.379581 |
|  | 40 | | | 0.052748 | | 0.15724 | | 0.986 | | -0.45079 | | | 0.556287 |
| 40 | 10 | | | -0.36964 | | 0.15724 | | 0.165 | | -0.87318 | | | 0.133895 |
|  | 20 | | | -0.17671 | | 0.15724 | | 0.686 | | -0.68024 | | | 0.326833 |
|  | 30 | | | -0.05275 | | 0.15724 | | 0.986 | | -0.55629 | | | 0.450791 |

| Multiple Comparisons (SUPPFIGS1H) | | | | |  | |  | | | |  | | |
| --- | --- | --- | --- | --- | --- | --- | --- | --- | --- | --- | --- | --- | --- |
| Dependent Variable: cell size | | | | |  | |  | | | |  | | |
| Tukey HSD | |  | |  | | |  | |  | | |  | |
| (I) day | | (J) day | | Mean Difference (I-J) | | | Std. Error | | Sig. | | | 95% Confidence Interval | |
|  |  | |  | | |  | |  | | Lower Bound | | | Upper Bound |
| 10 | 20 | | -162 | | | 158.075 | | 0.736 | | -585.71 | | | 261.71 |
|  | 30 | | -522.091* | | | 158.075 | | 0.01 | | -945.8 | | | -98.38 |
|  | 40 | | -2107.818* | | | 158.075 | | <0.0001 | | -2531.53 | | | -1684.11 |
| 20 | 10 | | 162 | | | 158.075 | | 0.736 | | -261.71 | | | 585.71 |
|  | 30 | | -360.091 | | | 158.075 | | 0.12 | | -783.8 | | | 63.62 |
|  | 40 | | -1945.818* | | | 158.075 | | <0.0001 | | -2369.53 | | | -1522.11 |
| 30 | 10 | | 522.091* | | | 158.075 | | 0.01 | | 98.38 | | | 945.8 |
|  | 20 | | 360.091 | | | 158.075 | | 0.12 | | -63.62 | | | 783.8 |
|  | 40 | | -1585.727* | | | 158.075 | | <0.0001 | | -2009.43 | | | -1162.02 |
| 40 | 10 | | 2107.818* | | | 158.075 | | <0.0001 | | 1684.11 | | | 2531.53 |
|  | 20 | | 1945.818* | | | 158.075 | | <0.0001 | | 1522.11 | | | 2369.53 |
|  | 30 | | 1585.727* | | | 158.075 | | <0.0001 | | 1162.02 | | | 2009.43 |
| * The mean difference is significant at the 0.05 level. | | | | | | |  | | | | | | |
